# The Identifying Depression Early in Adolescence Risk Stratified Cohort (IDEA-RiSCo): Rationale, Methods, and Baseline Characteristics

**DOI:** 10.3389/fpsyt.2021.697144

**Published:** 2021-06-21

**Authors:** Christian Kieling, Claudia Buchweitz, Arthur Caye, Pedro Manfro, Rivka Pereira, Anna Viduani, Maurício Anés, Lucas Battel, Silvia Benetti, Helen L. Fisher, Rakesh Karmacharya, Brandon A. Kohrt, Thais Martini, Sandra Petresco, Jader Piccin, Thiago Rocha, Luis Augusto Rohde, Fernanda Rohrsetzer, Laila Souza, Bruna Velazquez, Annabel Walsh, Leehyun Yoon, Zuzanna Zajkowska, Valentina Zonca, Johnna R. Swartz, Valeria Mondelli

**Affiliations:** ^1^Department of Psychiatry, Universidade Federal do Rio Grande do Sul (UFRGS), Porto Alegre, Brazil; ^2^Child and Adolescent Psychiatry Division, Hospital de Clínicas de Porto Alegre (HCPA), Universidade Federal do Rio Grande do Sul (UFRGS), Porto Alegre, Brazil; ^3^Medical Physics and Radioprotection Division, Hospital de Clínicas de Porto Alegre (HCPA), Porto Alegre, Brazil; ^4^Social, Genetic & Developmental Psychiatry Centre, King's College London, Institute of Psychiatry, Psychology & Neuroscience, London, United Kingdom; ^5^ESRC Centre for Society and Mental Health, King's College London, London, United Kingdom; ^6^Program in Neuroscience and Chemical Biology, Center for Genomic Medicine, Massachusetts General Hospital & McLean Hospital, Harvard University, Boston, MA, United States; ^7^Division of Global Mental Health, Department of Psychiatry, School of Medicine and Health Sciences, The George Washington University, Washington, DC, United States; ^8^ADHD Outpatient and Developmental Psychiatry Programs, Hospital de Clínicas de Porto Alegre (HCPA), Porto Alegre, Brazil; ^9^Department of Psychological Medicine, King's College London, Institute of Psychiatry, Psychology, London, United Kingdom; ^10^Department of Human Ecology, University of California, Davis, Davis, CA, United States; ^11^Biological Psychiatry Unit, IRCCS Istituto Centro San Giovanni di Dio Fatebenefratelli, Brescia, Italy; ^12^National Institute for Health Research Mental Health Biomedical Research Centre, South London and Maudsley NHS Foundation Trust and King's College London, London, United Kingdom

**Keywords:** depression, adolescence, risk score, cohort, neurobiology

## Abstract

**Background:** The characterization of adolescents at high risk for developing depression has traditionally relied on the presence or absence of single risk factors. More recently, the use of composite risk scores combining information from multiple variables has gained attention in prognostic research in the field of mental health. We previously developed a sociodemographic composite score to estimate the individual level probability of depression occurrence in adolescence, the Identifying Depression Early in Adolescence Risk Score (IDEA-RS).

**Objectives:** In this report, we present the rationale, methods, and baseline characteristics of the Identifying Depression Early in Adolescence Risk Stratified Cohort (IDEA-RiSCo), a study designed for in-depth examination of multiple neurobiological, psychological, and environmental measures associated with the risk of developing and with the presence of depression in adolescence, with a focus on immune/inflammatory and neuroimaging markers.

**Methods:** Using the IDEA-RS as a tool for risk stratification, we recruited a new sample of adolescents enriched for low (LR) and high (HR) depression risk, as well as a group of adolescents with a currently untreated major depressive episode (MDD). Methods for phenotypic, peripheral biological samples, and neuroimaging assessments are described, as well as baseline clinical characteristics of the IDEA-RiSCo sample.

**Results:** A total of 7,720 adolescents aged 14–16 years were screened in public state schools in Porto Alegre, Brazil. We were able to identify individuals at low and high risk for developing depression in adolescence: in each group, 50 participants (25 boys, 25 girls) were included and successfully completed the detailed phenotypic assessment with ascertainment of risk/MDD status, blood and saliva collections, and magnetic resonance imaging (MRI) scans. Across a variety of measures of psychopathology and exposure to negative events, there was a clear pattern in which either the MDD group or both the HR and the MDD groups exhibited worse indicators in comparison to the LR group.

**Conclusion:** The use of an empirically-derived composite score to stratify risk for developing depression represents a promising strategy to establish a risk-enriched cohort that will contribute to the understanding of the neurobiological correlates of risk and onset of depression in adolescence.

## Introduction

Major advances have been accomplished in healthcare through the identification of factors that increase or decrease the probability of an individual developing a negative outcome (1). In the field of cardiovascular medicine, for example, the identification of a set of risk factors has enabled the implementation of multiple preventative strategies that have ultimately translated into decreased burden of heart disease (2). A crucial aspect of this approach is the combination of multiple factors into one single, composite score—e.g., the Framingham Risk Score aggregates information from six variables to estimate the 10-year risk of coronary disease (3).

There is a dire need to reduce the burden associated with depressive disorders globally (4). Differently from other branches of medicine, however, research in the field of psychiatry and mental health has often examined a single risk factor at a time (e.g., poverty, child maltreatment, discrimination) in the effort to identify mechanisms associated with the disorder's pathophysiology. Despite unquestionable advances in the identification of individual markers of depression risk—notably the role of a positive family history of depression in increasing the probability of the disorder in the offspring—a broader, more comprehensive approach is likely to be required in the context of multifactorial disorders such as depression (5).

The incidence of depression peaks in adolescence (6), which implies not only a substantial disease-related burden early in life, but also an important window of opportunity for prevention. Universal approaches addressing entire groups of adolescents have been less successful than selective and indicated interventions focusing on those who are at high-risk because of the presence of either proximal risk factors or subclinical symptoms (7). To further advance targeted preventive interventions, however, an important challenge that remains is the characterization of who is at high risk, as well as which neurobiological, psychological, and environmental mechanisms are associated with the development of depression (8). Crucially, relying on single risk factors can be potentially misleading in the identification of high- and low-risk individuals, as, for instance, an adolescent with no family history of the disorder (frequently assigned as being at low risk) can actually be at an increased risk for developing depression due to the experience of other risk factors (e.g., childhood maltreatment) (9).

In fact, the ability to move beyond a binary approach to risk (i.e., absent/present) to incorporate a dimensional perspective is another opportune advantage of using composite scores. Most of the current samples in mental health research contrast cases and non-cases, with the latter usually defined by lack of a current psychiatric disorder. However, especially among younger individuals, non-cases may have a number of risk factors that make them likely to develop a disorder in the future, leading to a high degree of noise and heterogeneity in these typical designs. The use of risk scores derived from multiple risk factors therefore does not assume adolescents without the disorder as a homogenous group, allowing researchers to specifically focus on individuals at extremely high, but also at extremely low risk for developing depression.

In that sense, efforts have been proposed in terms of using composite scores to stratify risk, with great attention recently directed to the use of genetic information (10). Polygenic risk scores (PRS) are calculated as the sum of genetic risk variants for a specific trait or disorder weighted according to previous genome-wide association studies. Considering that non-genetic factors also contribute to the etiology of depression (5, 11), the case for what has been termed a “polysocial risk score” could also be made, modeling the combination of socio-environmental factors to capture individual-level risk of developing the disorder (12). As suggested by many PRS studies, a focus on extreme strata (e.g., below the lowest and above the highest deciles) could potentially allow for the characterization of more homogeneous groups.

**Figure 1:**
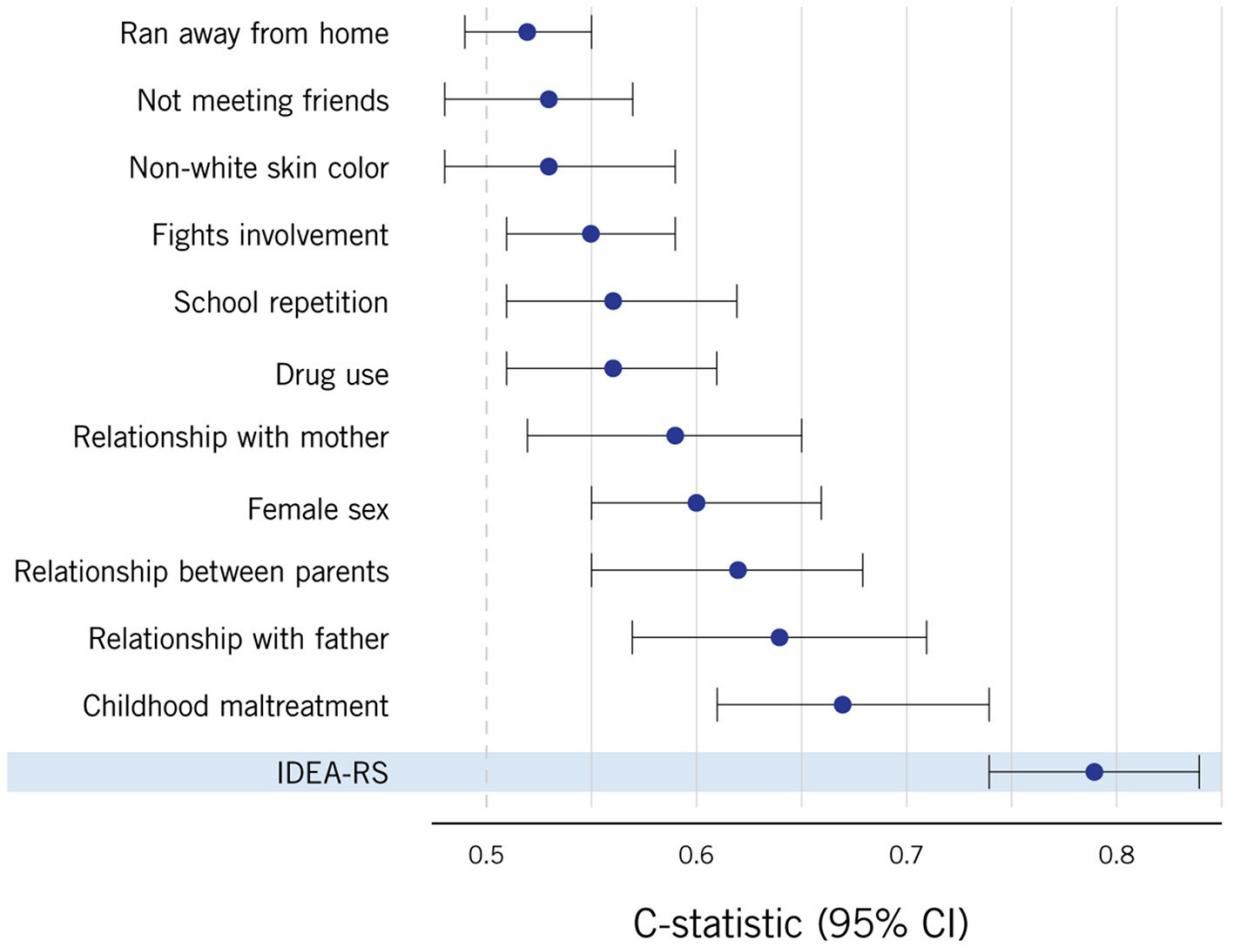
Discriminative ability of each individual Identifying Depression Early in Adolescence Risk Score (IDEA-RS) variable, as well as the combined statistic for the IDEA-RS in the last line. Generated using data from the Pelotas 1993 Birth Cohort (13).

As part of the Identifying Depression Early in Adolescence (IDEA) international consortium (8), our group has developed a composite score to estimate individual-level probability of developing major depression among Brazilian adolescents (13). The IDEA risk score (IDEA-RS) comprises only sociodemographic variables that can be easily obtained directly from the adolescent to facilitate translation into practice: biological sex, skin color, drug use, school failure, social isolation, fight involvement, relationship with mother, relationship with father, relationship between parents, childhood maltreatment, and ran away from home (Figure 1). Among 15-year-old adolescents in Brazil, the IDEA-RS exhibited good discriminative performance (C-statistic of 0.78) to parse individuals at high- and at low-risk for developing depression at age 18 (13). External validation indicated that the IDEA-RS was also able to predict the occurrence of depression in samples from other countries and continents (13–15).

As a further step, we here present the Identifying Depression Early in Adolescence Risk Stratified Cohort (IDEA-RiSCo), established to investigate neurobiological features associated with the risk of developing depression and with the presence of depression in adolescence, with a focus on immune/inflammatory and neuroimaging markers. Using the IDEA-RS as a tool for risk stratification, we recruited a new sample of adolescents enriched for low and high depression risk, as well as a group of adolescents with a currently untreated major depressive episode. Methods for phenotypic, peripheral biological samples, and neuroimaging assessments are described, as well as baseline clinical characteristics of the IDEA-RiSCo sample. Additionally, we present adolescents' perspectives on taking part in this study.

## Methods

### Ethics Approval

This study was approved by the Brazilian National Ethics in Research Commission (CAAE 50473015.9.0000.5327). Adolescents provided written assent and their primary caregivers written consent prior to entering the study. Approval for the school screening phase was obtained from the 1st Regional Education Bureau, in charge of public state schools in the city of Porto Alegre. All participants received feedback with findings from the diagnostic assessment and were referred for care in the Brazilian public health system if clinically indicated. Situations of imminent risk of self-harm or maltreatment were referred to emergency care or protective services following Brazilian legislation. Participants received no financial incentive for taking part in the study, but were compensated for expenses related to their participation (e.g., travel). Approval was also obtained from the Ethics Committee at King's College London for secondary data analysis for biological measures.

### Ascertainment and Group Assignment

In this report, we present cross-sectional data from the baseline stage of the IDEA-RiSCo study, following the STrengthening the Reporting of OBservational studies in Epidemiology (STROBE) guidelines (16). Individuals at low- and at high-risk for developing depression were identified using the IDEA-RS questions (Supplementary Material 1). The IDEA-RS was initially developed and validated on a sample of adolescents aged 15 years old to estimate the probability of a diagnosis of major depressive disorder at age 18 (13) in the Pelotas 1993 Birth Cohort Study (17). For the present study, 14 to 16-year-old adolescents (to resemble the developmental stage in which the IDEA-RS was originally devised) were screened in 101 public state schools located in the city of Porto Alegre, Brazil (see Supplementary Materials 2, 3 for detailed procedures). The answers to the questions were aggregated to create a continuous score (i.e., the IDEA-RS) for each adolescent who participated in the screening stage of the study. Using cut-offs for the IDEA-RS based on the Pelotas 1993 Birth Cohort Study (13), we *a priori* operationalized risk strata for recruitment of participants into the new cohort: low-risk (LR) adolescents were those scoring equal to or below the 20th percentile of the IDEA-RS; and high-risk (HR) adolescents were those scoring equal to or above the 90th percentile of the IDEA-RS. We allowed a larger stratum in the LR group as the absolute risk difference between the 10th and the 20th percentiles was minimal. Importantly, as the probability of depression is known to be higher in females in comparison to males, we opted to generate sex-specific IDEA-RS in order to guide the recruitment of this risk-enriched sample. According to IDEA-RS in Pelotas, the probabilities of depression for the 20th and the 90th percentiles were 1.87 and 8.39% for girls and 1.12 and 3.37% for boys. Of note, these estimates refer to the probability of presenting a depressive episode exactly at age 18 years, as in the Pelotas 1993 Cohort Study only the point-prevalence of a current unipolar depressive episode was assessed. This means that the lifetime probabilities of MDD are likely higher for all groups.

In addition to the LR and HR groups, we also recruited a third group of adolescents with major depressive disorder (MDD). To allow for two-by-two comparisons between groups, adolescents with MDD were also required a score equal to or above the 90th percentile of the IDEA-RS. Thus, LR and HR groups were similar in showing no lifetime history of any depressive disorder, but markedly different regarding the IDEA-RS. Conversely, HR and MDD groups were similar regarding IDEA-RS, but while HR participants showed no evidence of depression at any time, those in the MDD group had to be in a current unipolar depressive episode at the time of the assessment.

To optimize the recruitment process and increase the probability that diagnostic criteria for depression were met in the MDD group, but not in the LR and HR groups, during the school screening adolescents also completed the Patient Health Questionnaire—adolescent version (PHQ-A) (18). Adolescents with a PHQ-A ≤ 6 were considered for further assessment for the LR/HR groups, and those with a PHQ-A ≥ 10 for the MDD group. Importantly, PHQ-A cutoffs were necessary but not sufficient for group assignment, as, for instance, the absence of a lifetime history of depressive disorders was also required for the LR/HR groups, and this was only determined during clinical assessment.

Based on school screening information, participants meeting criteria for further assessment were invited to the Clinical Research Center at Hospital de Clínicas de Porto Alegre (HCPA). Clinical assessment was conducted by board-certified child and adolescent psychiatrists who individually interviewed both the adolescent and their primary caregiver and were unaware of the participant's risk group status. Absence of a lifetime history of depressive disorders (including dysthymia) for the LR and HR groups and presence of a current depressive episode for the MDD group were determined using the Brazilian Portuguese translation of the Schedule for Affective Disorders and Schizophrenia for School-Age Children-Present and Lifetime Version (K-SADS-PL) (19). Clinicians received prior inter-reliability training on the K-SADS-PL, and for each participant a clinical formulation and best estimate diagnoses were generated and subsequently reviewed by an experienced child and adolescent psychiatrist (CK) to confirm diagnoses and assure uniformity in participant assignment. Participants in all three groups were excluded if they met lifetime diagnostic criteria for autism spectrum disorder, bipolar disorder, eating disorders, post-traumatic stress disorder, schizophrenia, or substance use disorders. Additional exclusion criteria are listed in Supplementary Material 2.

### Phenotypic Assessment

Youth assigned to LR, HR, or MDD groups underwent further phenotypic assessment. Comorbid diagnoses were assessed using the K-SADS-PL (19). Whereas the module on mood disorders was applied to both adolescents and caregivers, other domains were assessed primarily using information obtained from adolescents (anxiety, obsessive-compulsive, trauma-related, eating, and substance use disorders) or caregivers (schizophrenia/psychosis and neurodevelopment/disruptive disorders). Adolescents' IQ was estimated using the Wechsler Abbreviated Scale of Intelligence (WASI) (20, 21). Caregivers were asked about the adolescent's family history of depression—information was collected on parents, grandparents, and siblings and summarized in a family liability index that estimates the proportion of affected family members, adjusting for relatedness (22). Pubertal stage was determined by adolescent self-report using the Tanner Puberty Staging Scale (23). Further psychological and socio-environmental assessments included self- and clinician-based instruments as described in Table 1, Supplementary Material 4.

**Table 1:** Domains and instruments used for phenotypic characterization of the IDEA-RiSCo sample.

**Domain**	**Instrument**
**Adolescents**	
Overall psychopathology	DSM-5 Self-Rated Level 1 Cross-Cutting Symptom Measure, Child (CCSM-C) (24, 25)
Depression	Mood and Feelings Questionnaire—Child (MFQ-C) (26, 27)
Anhedonia	Snaith-Hamilton Pleasure Scale (SHAPS) (17, 28)
Irritability	Affective Reactivity Index—Child (ARI-C) (29, 30)
Suicidality	Columbia-Suicide Severity Rating Scale (C-SSRS) (31)
Anxiety	Spence Children's Anxiety Scale (SCAS-C) (32, 33)
Insomnia	Insomnia Severity Index (ISI) (34, 35)
Reflexive functioning	Reflective Functioning Questionnaire for Youth (RFQY) (36, 37)
Resilience	Adapted Resilience Scale (ARS)^*^ (38, 39)
Positive attributes	Youth Strengths Inventory—Adolescent (YSI-A) (40, 41)
Parental bonding (separate measures for mother and father)	Parental Bonding Instrument (PBI) (42)
Maltreatment/trauma history	Child Trauma Questionnaire (CTQ) (43, 44)
Recent life events	Life Events Questionnaire (LEQ)^*^ (45)
Physical activity	Patient-Centered Assessment and Counseling for Exercise Plus Nutrition^*^ (PACE+) (46)
**Primary caregivers**	
Overall psychopathology	DSM-5 Self-Rated Level 1 Cross-Cutting Symptom Measure, Parent (CCSM-P) (24)
Depression	Mood and Feelings Questionnaire—Parent (MFQ-P) (26, 27)
Irritability	Affective Reactivity Index—Parent (ARI-P) (29, 30)
Anxiety	Spence Children's Anxiety Scale—Parent (SCAS-P) (32, 33)
Positive attributes	Youth Strengths Inventory—Parent (YSI-P) (40, 41)
Socioeconomic status	Brazil socioeconomic classification index (ABEP) (47)
Caregiver's depression	Mood and Feelings Questionnaire—Adult (MFQ-A) (26, 27)
**Combined information (adolescent** **+** **caregiver)**	
Depression	Children's Depression Rating Scale Revised (CDRS-R) (48, 49)
Clinical global impression	Clinical Global Impression (CGI) (50)
Global functioning	Children's Global Assessment Scale (CGAS) (51, 52)

* *Instruments for which we performed the translation into Brazilian Portuguese following the steps described in Supplementary Material 4. IDEA-RiSCo, Identifying Depression Early in Adolescence Risk-Stratified Cohort*.

Anthropometric measurements were performed right after the clinical evaluation. Axillary temperature (°C) was measured using an electronic thermometer. Weight (kg) was measured using an electronic scale, with individuals wearing light clothes and without shoes. Height (cm) was measured using a stadiometer. Waist circumference (cm) was measured with a non-stretching tape at the midpoint between the iliac crest and the lowest rib margin.

### Collection of Blood and Saliva Samples

**Figure 2:**
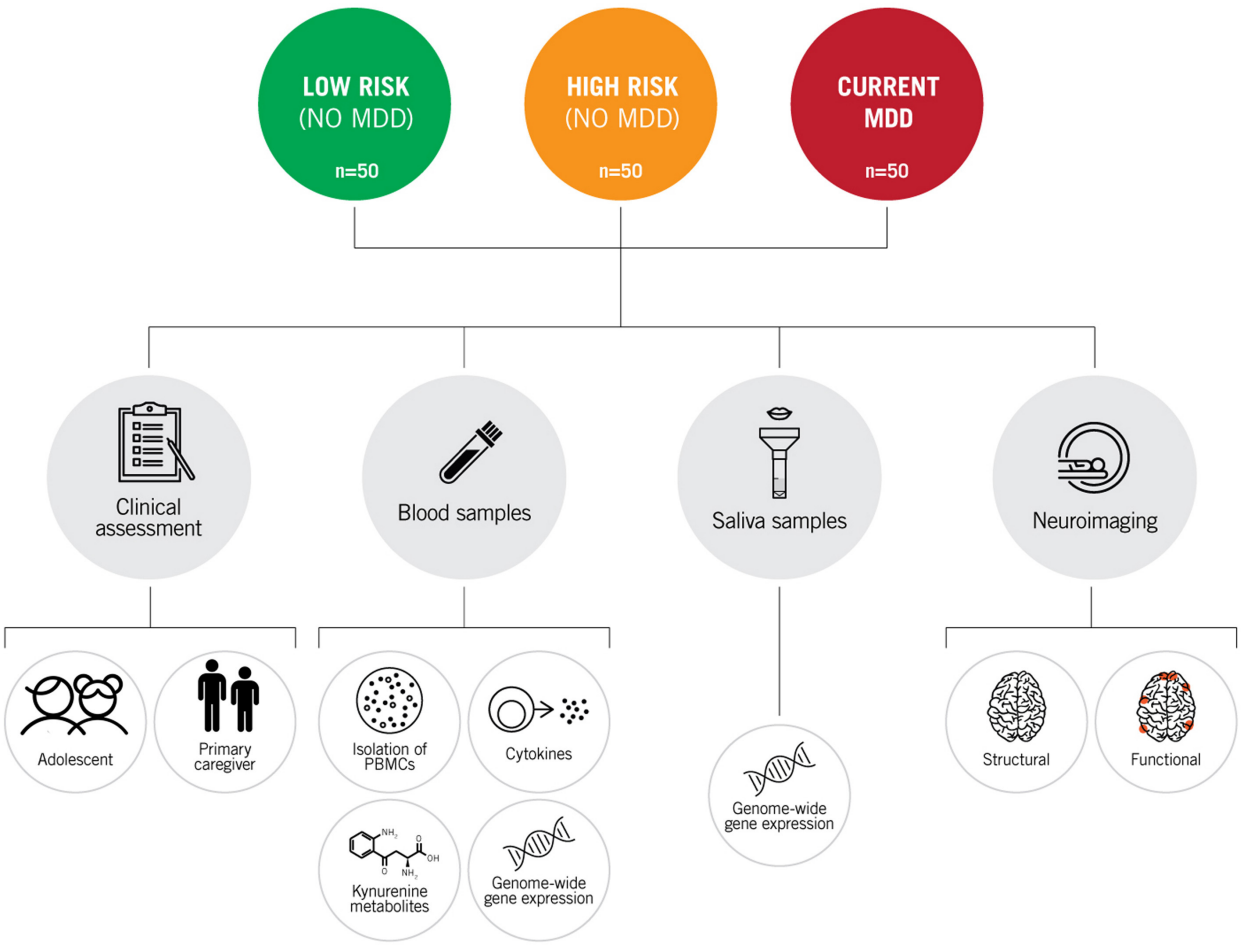
Simplified graphic representation of the assessments and analyses performed in the IDEA-RiSCo cohort, including blood and saliva analyses such as isolation of peripheral blood mononuclear cells (PBMCs) and determination of cytokines and kynurenine metabolites and genome-wide gene expression. IDEA-RiSCo, Identifying Depression Early in Adolescence Risk-Stratified Cohort; MDD, major depressive disorder; MRI, magnetic resonance imaging; fMRI, functional magnetic resonance imaging.

On the same day of clinical/phenotypic assessment, once the risk/MDD status was ascertained, participants underwent collection of blood and saliva samples (Figure 2). Only participants for whom blood and saliva samples were successfully collected were included in the cohort.

Briefly, procedures included a previous instruction not to change their eating habits the day before the blood and saliva collection, and to take any medications as usual. Participants were also required to avoid excessive fasting (over 24 h); to avoid intake of any kind of food, natural water, coffee, tea, juice, milk, or other drinks at least 2 h before the collection; and to avoid smoking or chewing gum during the period between awakening and sample collection. The following samples were collected, processed, and stored at −80°C: serum from whole blood (6.0 mL of blood using a vacutainer tube without any anticoagulant); plasma from EDTA whole blood (6.0 mL of blood using a K3EDTA anticoagulant tube); RNA (2.5 mL of blood using PAXGene tubes, PreAnalitix, Qiagen/BD Company). Peripheral blood mononuclear cells (PBMC) were collected from whole blood (4.0 mL of blood collected in 2 Vacutainer EDTA tubes) by the density gradient centrifugation method using Histopaque®-1077 reagent (Sigma-Aldrich) according to manufacture instructions. The cells were kept frozen in liquid nitrogen with a cryoprotectant solution (bovine fetal serum F4135-Gibco and 10% DMSO-D2650-Sigma Aldrich). Saliva samples were collected using Oragene RNA tubes (RE-100) supplied by DNA Genotek (Ottawa, Ontario, Canada). A total of 2.0 mL of unstimulated saliva was collected by directly spitting into the tubes; once collected, Oragene RNA tubes were stored at −20°C.

All samples were shipped using a courier specialized for transferring biological samples. Four serum, four plasma, and two PBMC cryovials were sent in a single batch to The Maurice Wohl Clinical Neuroscience Institute Laboratory at King's College London, United Kingdom. One PAXGene tube and saliva samples were sent in two batches to IRCCS Istituto Centro San Giovanni di Dio Fatebenefratelli in Brescia, Italy. The remaining two serum and plasma cryovials and one PAXGene tube were kept as a backup in Brazil.

### Magnetic Resonance Imaging

Magnetic resonance imaging (MRI) was performed on the same day, following collection of blood and saliva samples. Only participants who were able to successfully complete the entire MRI procedure were included in the cohort. Both structural and functional images were acquired on a 3T Ingenia scanner (Koninklijke Philips N.V., The Netherlands), software version 5.3.1, at Hospital de Clínicas de Porto Alegre.

**Figure 3:**
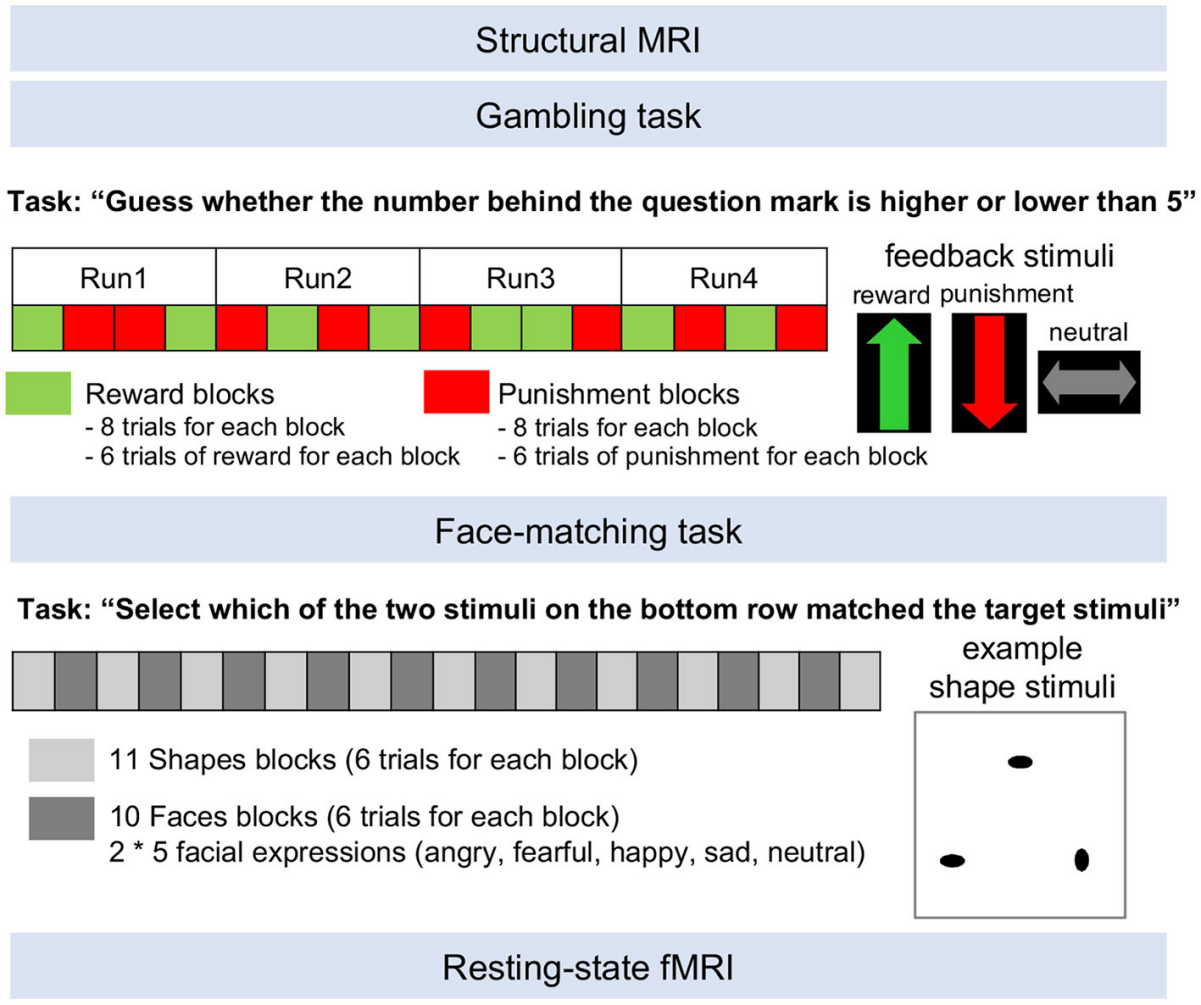
MRI data acquisition procedure. After structural images were acquired, functional images were acquired during a gambling task, face-matching task, and resting-state. The gambling task consisted of counterbalanced presentation of reward and punishment blocks. The face-matching task consisted of counterbalanced presentation of shapes and faces blocks. Feedback stimuli for the gambling task and an example shape stimulus for the face-matching task are presented. During the resting-state fMRI scan, participants were instructed to let their minds wander.

Before entering the MRI suite, participants were asked to remove all metal objects from their body (e.g., earrings, piercings, rings, watches). They received instructions regarding scanning procedures (including the request to keep their head still during the scan) and scanning duration. A 30-s demonstration for each task was provided. Finally, they were informed about loud banging noises during scanning, and that communication with the experimenter would be possible at any time during the scan. Once they entered the MRI room, participants were positioned in the scanner. Images were acquired in the same order for every participant—structural, gambling task, face-matching task, and resting-state (Figure 3; see Supplementary Material 5 for data acquisition parameters).

#### Tasks

The gambling task was adapted from Barch et al. (53) and translated into Brazilian Portuguese. The task was to guess whether the number behind a question mark was higher or lower than 5 by using two one-button boxes with the left and right index fingers. After each guess, participants received pre-determined feedback consisting of reward (i.e., correct guess), punishment (i.e., incorrect guess), and neutral feedback (i.e., the number is 5). The task included four runs, each with 2 blocks consisting primarily of reward trials (i.e., 6 out of 8 trials) and blocks consisting primarily of punishment trials (i.e., 6 out of 8 trials) in each run. The task consisted of 4 runs with different orders of reward and punishment blocks, which were counterbalanced across participants. Each block took 28 s and consisted of 8 trials, which contained a question mark (1.5 s) and feedback (1 s). Participants conducted at least 10 practice trials before the actual task.

The face-matching task was adapted from Hariri et al. (54) and translated into Brazilian Portuguese. During the task, participants viewed a trio of faces or shapes and had to select which of two stimuli on the bottom row matched the target stimuli on the top row by pressing a button with their left or right index finger. This task included counterbalanced presentation of 10 face blocks, including 5 facial expressions (i.e., angry, fearful, happy, sad, and neutral faces) and 11 shapes blocks. Face and shape blocks were alternatively presented and the order of face blocks was counterbalanced across participants. Each block included 6 trials. Face blocks included 2 blocks of 5 facial expressions (i.e., angry, fearful, happy, sad, neutral). Each block took 26 s and consisted of 6 trials with 2 s of stimuli presentation.

#### Task-based fMRI Data Analysis

**Figure 4:**
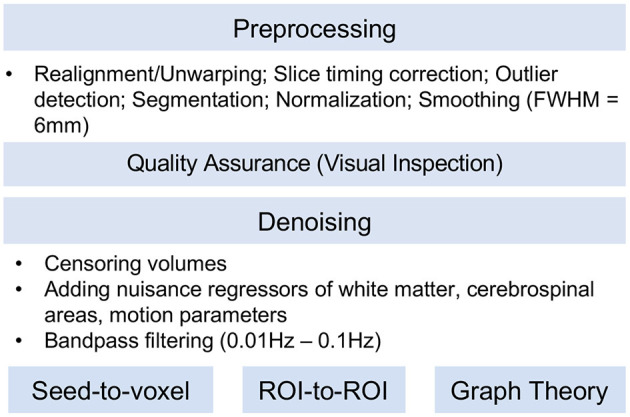
Pre-processing pipeline for resting-state fMRI. Data will be preprocessed with the sequence of realignment/unwarping, slice timing correction, head movement outlier detection, segmentation, normalization, and smoothing with 6 mm of Full width at half maximum (FWHM). Each subject's images will be visually inspected for quality control. Denoising will be performed by volume censoring with the conservative criteria (i.e., the threshold of a global-signal z-value of 3 or 0.5 mm volume-to-volume motion), adding nuisance regressors of white matter, cerebrospinal areas, and motion parameters, and bandpass filtering to a 0.01–0.1 Hz window. For the statistical analysis, seed-to-voxel, ROI-to-ROI, and graph theory analysis will be implemented. FWHM, Full width at half maximum; ROI, Region of Interest.

After preprocessing (Supplementary Material 6), we estimated generalized linear models (GLM) to examine neural activity and connectivity during reward processing (i.e., reward vs. punishment) and emotional face processing (i.e., angry faces vs. shapes, fearful faces vs. shapes, happy faces vs. shapes, sad faces vs. shapes, and neutral faces vs. shapes), and we generated contrast maps (Figure 4). The contrast maps of each individual will be carried forward into group-level random-effects models and will be used to examine differences in neural activity between the LR, HR, and MDD groups in future research papers.

#### Resting-State fMRI Data Analysis

The resting-state functional connectivity (rsFC) images were preprocessed and denoised using the CONN toolbox (https://web.conn-toolbox.org). In future papers, we plan to conduct three types of analyses to examine differences in rsFC between the LR, HR, and MDD groups: (1) seed-based connectivity analysis that examines the connectivity between a seed region (e.g., amygdala, posterior cingulate cortex) and other regions in the whole brain, (2) ROI-to-ROI analysis that examines the connectivity of all nodes within a specific network, and (3) graph theory analysis that examines the topological properties of a network (e.g., how much a particular node is efficiently connected with other nodes of the network) (Figure 4).

### Sample Size Calculation

One of the major goals for this study is to examine both concurrent and prospective (in planned longitudinal follow-ups that are underway) associations between risk status, depression symptoms, and neurobiological features. In prior work (55), an IDEA investigator had examined differences in threat-related amygdala function in adolescents at high familial risk for depression compared to those at lower risk, and with high exposure to recent life stress compared to low exposure to recent life stress. In that research, models that included age, family history, and recent life stress as predictors explained 11% total variance in amygdala function. Thus, for the IDEA-RiSCo sample, we conducted a power analysis using an expected effect size of partial η^2^ = 0.10. Assuming this effect size and an F-test with 3 groups stratified by sociodemographic risk and MDD status, we estimated we would need at least 90 participants (30 in each group) to identify an effect of this size with at least 80% power. Additionally, prior research has shown that neural activity predicts depression/internalizing symptoms with effect sizes of partial *r*^2^ = ~0.05–0.30 (29, 56–59). We computed a power analysis using G^*^Power based on partial *r*^2^ = 0.10 and obtained a required sample size of 73 to achieve 80% power to detect significant associations between neural activity and continuously-measured depression symptoms. Based on these power analyses, we determined a sample size of at least 90 participants would be required to test our primary hypotheses. We also assumed there would be ~10% data loss in the MRI data due to quality control procedures, which would require a total sample of 100 participants to achieve a final sample of 90 participants meeting all quality control criteria. Because we also planned to follow participants longitudinally and assumed some loss of data due to attrition and MRI quality control at the second longitudinal scan, we determined our final sample size for the baseline data collection to be 150 participants (50 LR, 50 HR, and 50 MDD).

### Data Management and Statistical Analyses

All clinical data were collected and managed using the Research Electronic Data Capture (REDCap) system hosted at Hospital de Clínicas de Porto Alegre (60, 61).

Sample characteristics are presented using descriptive statistics, Kruskal-Wallis, two proportion *Z*-test, and network analysis. The Kruskal-Wallis non-parametric test was used for mean comparisons, as all distributions of the instruments were non-normal. Two-proportion *Z*-tests were used to compare the proportions of risk score variables in the Porto Alegre vs. Pelotas samples (62). Network analysis was performed using the Mixed Graphic Model, which estimates networks from data with dichotomous, categorical, discrete and continuous variables (62). All statistical analyses were performed using R 3.6.1 (R Foundation for Statistical Computing, Vienna, Austria) through RStudio. A *p* < 0.05 was considered the threshold for statistical significance. The Tidyverse package (63) was used for data manipulation. The ggplot2 package was used for plotting figures (64). The “bootnet” package (65) and “mgm” method (corresponding to the Mixed Graphic Model) were used for network analysis. This model allows simultaneous analysis of different types of variables (e.g., categorical, dichotomized, and continuous). The “cor_auto” method, which automatically computes an appropriate correlation matrix for polychoric and polyserial correlations, was used to calculate correlations between variables. To visualize the networks, the qgraph package with the layout = “string” function was used, corresponding to the Fruchterman-Reingold algorithm for approximation of variables. Network structure and connectivity were compared with the Network Comparison Test (NCT) (66).

### Qualitative Component

Qualitative data collection on the study experience is an extension of a broader IDEA qualitative study on feasibility and acceptability of early detection of depression among adolescents in global settings (67). Qualitative interviews aimed to explore the experience of adolescents diagnosed with depression while taking part in the clinical evaluation. These participants were sampled by convenience, as the recruitment began at the final stages of the IDEA-RiSCo baseline assessment: the last 10 included adolescents who met criteria for a formal DSM-5 diagnosis of depression were invited to participate. They were first approached by the interviewers after the clinical evaluation and were invited to participate in two semi-structured interviews: one immediately after the clinical evaluation and the second 2 weeks later. This interview focused on understanding the adolescents' reaction to receiving a diagnosis of depression, but also explored the experience of participating in the clinical evaluation, having their blood and saliva collected and doing the fMRI, and their comprehension of the study's aims and objectives. Both interviews were audio recorded and later transcribed. The final analysis included 8 adolescents, as two were excluded due to incompleteness of their second interview.

One-on-one interviews were conducted in Brazilian Portuguese by two researchers (AV and SB, who had previous training and experience in qualitative research) and took place in a private room in the same setting as the remainder of the research protocol. Coding was done by both researchers using Framework Analysis (FA) (40) and this process was supervised by a third senior researcher (CK). The creation of the codes was inductive—we used line-by-line coding of two initial interviews to create a framework of codes that was later adapted and expanded until no new codes emerged (68). Additionally, constant comparison methods (69) and discussions with the research team were used to refine and create the final codebook. The full dataset was coded by two researchers using NVivo version 12 (70). Inter-rater reliability was assessed using Cohen's Kappa with 0.7 indicating adequate agreement (71). Afterward, code queries were generated in NVivo, and code summaries were written to capture adolescents' perspectives and experiences. Results highlight the main aspects of participation, presenting the number of adolescents who endorsed such views and following the steps of the described research protocol.

## Results

### The IDEA-RS in Porto Alegre and Its Comparison to Pelotas

Between July 2018 and November 2019, 7,720 adolescents (54.93% females) were screened in 101 schools (for details, see Supplementary Material 3). A comparison of the IDEA-RS in Porto Alegre and Pelotas, where the risk score was originally developed, indicated a higher average probability of developing a depressive episode within 3 years in Porto Alegre (5.30%) in relation to what was observed in Pelotas (3.39%). Supplementary Material 7 shows the probability of depression in 3 years for girls and boys in Porto Alegre and Pelotas.

**Figure 5:**
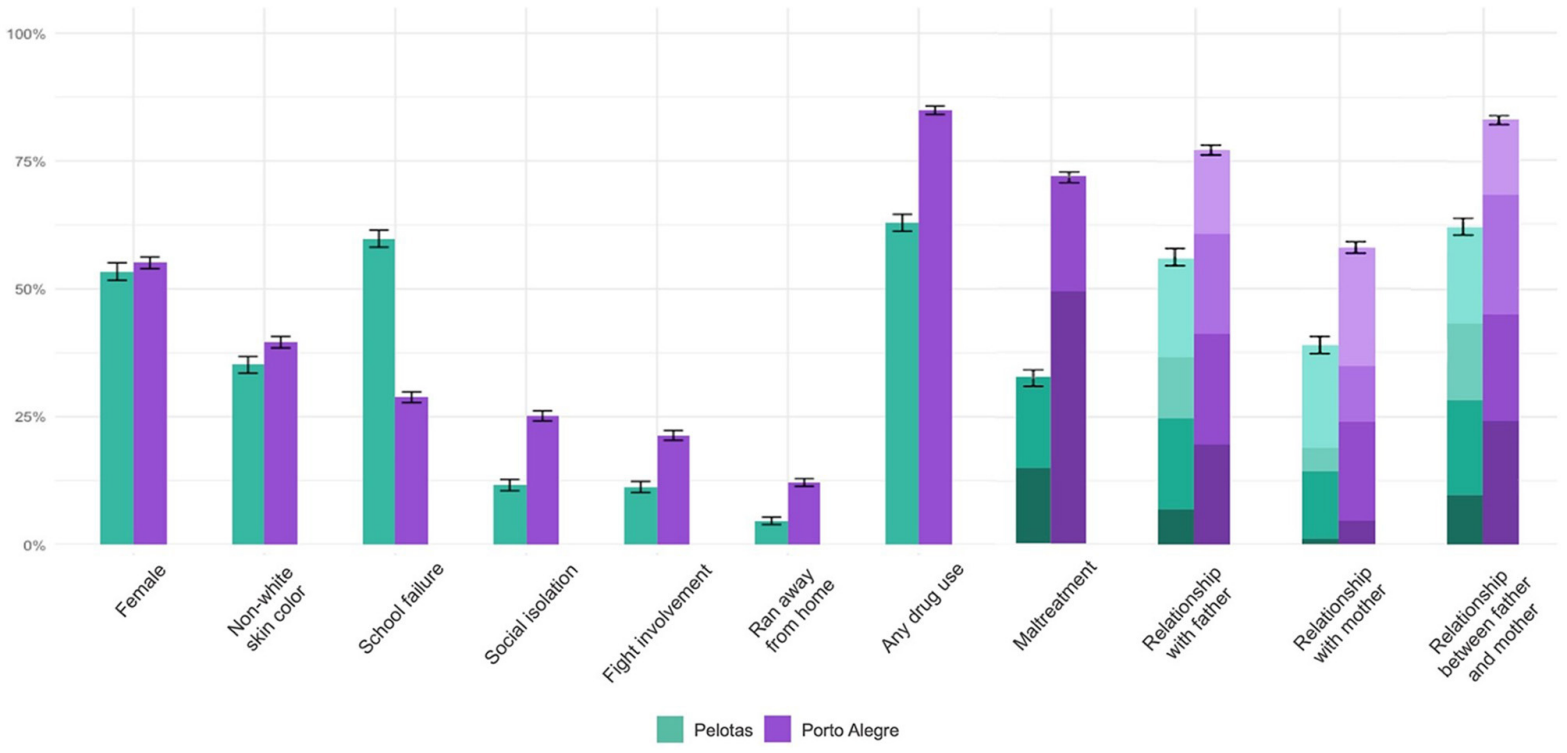
Prevalence of risk variables in Pelotas (2008) vs. Porto Alegre (2018–9). Proportions do not reach 100% because the figure only shows responses that indicate risk for the variables (i.e., non-involvement in fights or non-use of drugs are not shown). 95% confidence intervals are shown in dichotomous variables. In ordinal variables darker shades (bottom) indicate greater severity. The maltreatment variable is divided into “severe” (darkest green or purple) and “probable,” while relationship variables are divided into “bad” (darkest green or purple), “regular,” “good,” and “very good.” *n* = 3,290 (Pelotas) and *n* = 7,229 (Porto Alegre) adolescents with complete Identifying Depression Early in Adolescence risk score (IDEA-RS) questionnaires.

Individual IDEA-RS variables were more prevalent in Porto Alegre than in Pelotas (Figure 5), with two exceptions: biological sex, which was not significantly different in the two samples, and school failure, which was more prevalent in Pelotas. The higher prevalence of school failure could be expected in the population-based Pelotas sample, as opposed to the school-based Porto Alegre sample, which included only students around the expected grade for age.

**Figure 6:**
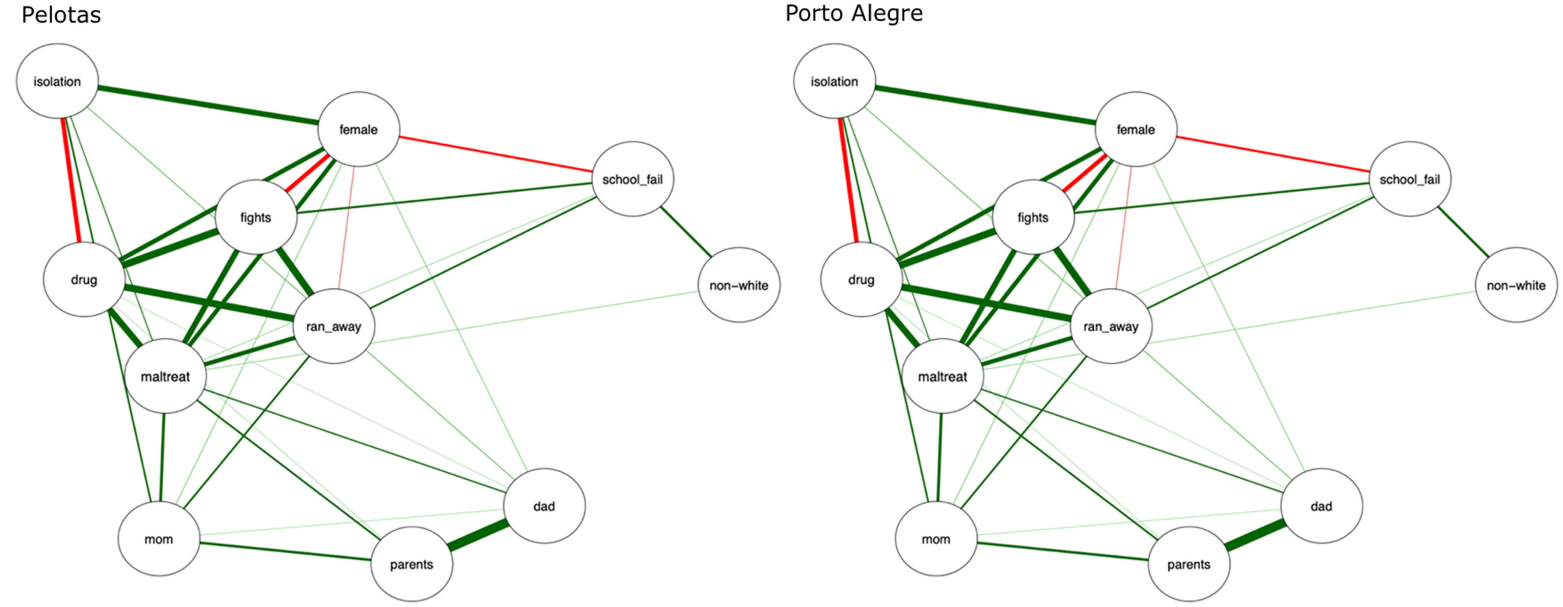
Network analysis of variables that are part of the IDEA-RS by cohort. Structure of Pelotas and Porto Alegre Identifying Depression Early in Adolescence risk score (IDEA-RS) networks with 11 nodes, each representing one of the risk score's variables. The green linkage lines between variables represent positive correlations. The red linkage lines represent negative correlations. Thicker lines represent greater strength of the correlation, which is based on the weighted network analysis. non-white, non-white skin color; drug, any drug use; school_fail, history of school failure; isolation, social isolation; fights, fight involvement; mom, relationship with mother; dad, relationship with father; parents, relationship between parents; maltreat, childhood maltreatment; ran_away, ran away from home.

To further explore potential similarities and differences of the IDEA-RS in Pelotas vs. Porto Alegre, we performed a network analysis to assess the associations among variables in both samples. We observed a similar pattern of positive and negative associations between the 11 nodes in the two networks (Figure 6). There was no evidence of significant differences in terms of connectivity (summarized by global strength, which is taken as the weighted absolute sum of all edges in the network) (72) or structure (calculated by the distance measure *M*, which is based on the maximum difference in edge weights of the observed networks) (73), suggesting comparability between the Pelotas and the Porto Alegre samples. A detailed description of the network analysis results can be found in Supplementary Material 8.

### Characteristics of Adolescents Included in the IDEA-RiSCo

**Figure 7:**
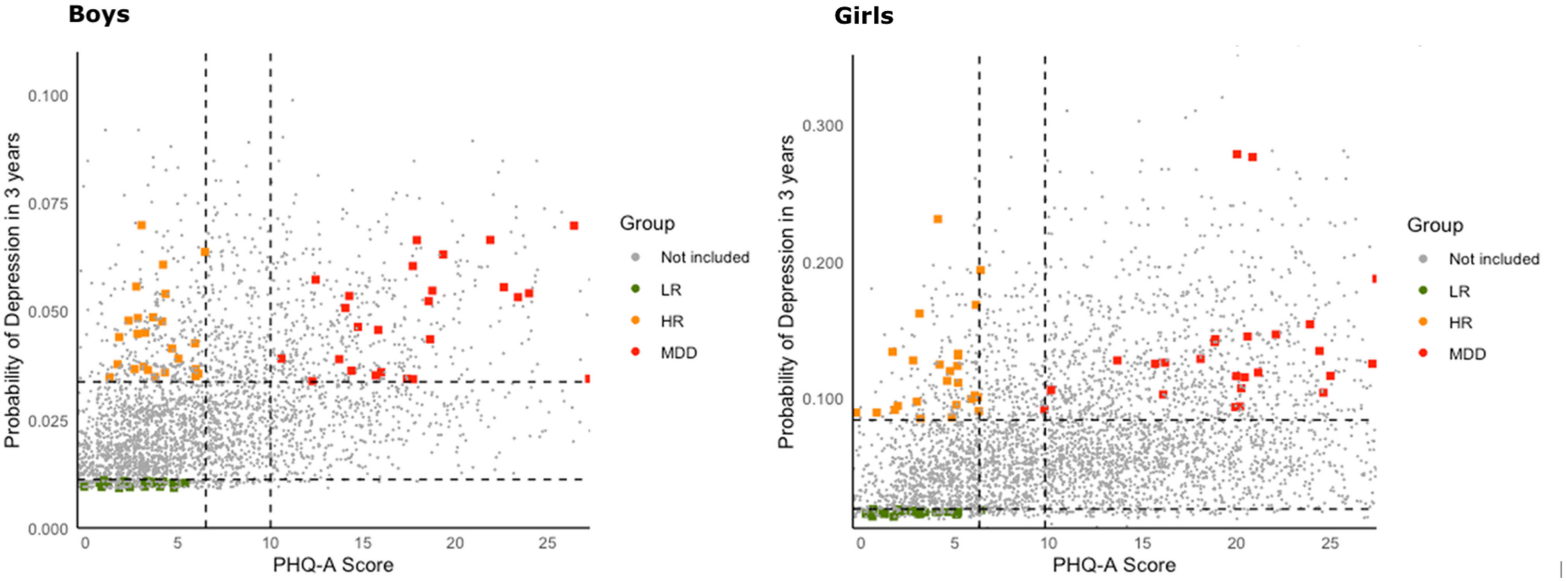
Adolescents screened at schools and included in the Identifying Depression Early in Adolescence Risk Stratified Cohort (IDEA-RiSCo). Vertical dotted lines show the Patient Health Questionnaire—adolescent version (PHQ-A) cutoffs, and horizontal dotted lines show the Identifying Depression Early in Adolescence risk score (IDEA-RS) cutoffs. Low risk (LR) adolescents appear in the lower left quadrant (PHQ-A ≤ 6 and IDEA-RS ≤ 20th percentile); high risk (HR) adolescents in the upper left quadrant (PHQ-A ≤ 6 and IDEA-RS≥90th percentile); and adolescents with current major depressive disorder (MDD), in the upper right quadrant (PHQ-A≥10 and IDEA-RS≥90th percentile). Gray dots representing the students who did not meet inclusion criteria are spread over all quadrants.

School screening in Porto Alegre confirmed higher IDEA-RS for girls (7.34%) in comparison to boys (2.78%). The mean PHQ-A score was 9.52, with higher scores also observed for girls (11.51 vs. 7.07 in boys). To reach the target sample size, 260 clinical assessments were conducted at Hospital de Clínicas de Porto Alegre. The distribution of IDEA-RS and PHQ-A for all boys and girls screened in schools appears in Figure 7, which also shows the 150 adolescents included in the IDEA-RiSCo sample. Following study design, both LR and HR adolescents exhibited lower mean PHQ-A scores in comparison to those with MDD. Likewise, mean IDEA-RS was lower for the LR in comparison to HR and MDD groups. In terms of age, there was a small but significant difference between groups, with the LR group being slightly younger than the HR and MDD groups. Detailed statistics are presented in Table 2.

**Table 2:** Phenotypic characteristics of the IDEA-RiSCo sample.

	**Low risk (*n* = 50)**	**High risk (*n* = 50)**	**MDD (*n* = 50)**	**Group differences^b^**
	**Mean (SD)^a^**	**Mean (SD)^a^**	**Mean (SD)^a^**	
Adolescent self-report				
Age (years)	15.36 (0.81)	15.76 (0.83)	15.80 (0.75)	LR < (HR = MDD)
IDEA-RS (%)	1.33 (0.32)	8.21 (4.61)	9.24 (5.60)	LR < (HR = MDD)
PHQ-A	2.82 (1.53)	3.96 (1.59)	18.82 (4.48)	(LR = HR) < MDD
MFQ-C	6.74 (4.84)	12.8 (8.36)	41.2 (11.11)	LR < HR < MDD
SHAPS	5.66 (3.93)	10.66 (5.54)	14.52 (6.79)	LR < HR < MDD
ARI-C	1.54 (2.07)	3.18 (2.73)	8.4 (3.83)	LR < HR < MDD
C-SSRS (lifetime)	0.00 (0.00)	1.72 (3.91)	14.64 (5.81)	(LR = HR) < MDD
SCAS-C	23.02 (11.03)	25.46 (11.27)	47.66 (20.45)	(LR = HR) < MDD
ISI	2.44 (3.12)	3.44 (2.81)	10.96 (4.72)	(LR = HR) < MDD
RFQ-Y	9.94 (1.58)	9.74 (1.57)	8.94 (1.87)	LR > MDD
YSI-A	27.8 (3.75)	25.7 (5.52)	21.7 (5.51)	(LR = HR) > MDD
PBI (mother)				
Care	31.69 (5.42)	26.60 (6.79)	21.66 (8.60)	LR > HR > MDD
Overprotection	13.08 (5.76)	16.08 (5.67)	18.94 (8.25)	LR < (HR = MDD)
PBI (father)				
Care	29.90 (6.36)	21.13 (7.79)	14.56 (8.56)	LR > HR > MDD
Overprotection	10.38 (5.58)	14.36 (6.86)	18.74 (10.05)	LR < (HR = MDD)
CTQ	29.16 (3.35)	38.08 (8.23)	51.56 (13.16)	LR < HR < MDD
LEQ				
Positive events	1.00 (0.93)	0.92 (0.99)	0.76 (0.94)	
Neutral events	0.52 (0.68)	0.46 (0.84)	0.70 (1.16)	
Negative events	1.24 (1.27)	1.58 (1.39)	3.04 (2.06)	(LR = HR) < MDD
ARS	46.80 (4.80)	43.40 (7.24)	36.44 (9.44)	(LR = HR) > MDD
PACE+	3.17 (2.31)	2.55 (2.08)	2.11 (2.06)	
Caregiver report				
MFQ-P (parent on child)	6.26 (8.37)	8.64 (7.74)	20.46 (12.30)	(LR = HR) < MDD
ARI-P	1.24 (2.44)	2.58 (3.39)	6.68 (4.82)	(LR = HR) < MDD
SCAS-P	13.62 (11.74)	14.00 (9.61)	21.16 (12.54)	(LR = HR) < MDD
YSI-P	39.30 (7.40)	37.56 (6.77)	32.24 (7.93)	(LR = HR) > MDD
ABEP	31.88 (9.78)	25.27 (7.63)	26.78 (9.28)	LR > (HR = MDD)
MFQ-A (parent self-report)	12.34 (14.59)	15.68 (13.01)	20.82 (14.22)	LR < MDD
Family liability index	0.13 (0.18)	0.20 (0.16)	0.24 (0.21)	LR < (HR = MDD)
Combined (adolescent + caregiver)				
CDRS-R	19.3 (2.85)	22.6 (5.44)	50.94 (9.79)	(LR = HR) < MDD
CGI-S	1.32 (0.55)	1.82 (0.75)	3.76 (0.66)	(LR = HR) < MDD
CGAS	90.00 (6.67)	83.52 (8.57)	55.52 (8.78)	LR > HR > MDD
Other				
WASI (IQ)	90.06 (10.16)	88.04 (8.57)	88.64 (9.76)	
Body mass index	22.61 (5.46)	22.4 (4.84)	22.75 (3.87)	
Body temperature	35.88 (0.59)	36.01 (0.51)	36.07 (0.62)	
Afternoon evaluations, *n* (%)	30 (60.00)	31 (62.00)	30 (60.00)	

a *Unless noted as n (%)*.

b *For a p < 0.05, comparisons between low risk (LR) vs. high risk (HR), LR vs. major depressive disorder (MDD), and HR vs. MDD, as indicated. ABEP, Brazil socioeconomic classification index; ARI-C, Affective Reactivity Index–Child; ARI-P, Affective Reactivity Index–Parent; ARS, Adapted Resilience Scale; CDRS-R, Children's Depression Rating Scale Revised; CGAS, Children's Global Assessment Scale; CGI-S, Clinical Global Impression–Severity scale; C-SSRS, Columbia-Suicide Severity Rating Scale; CTQ, Child Trauma Questionnaire; IDEA-RS, Identifying Depression Early in Adolescence Risk Score; IDEA-RiSCo, Identifying Depression Early in Adolescence Risk-Stratified Cohort; ISI, Insomnia Severity Index; LEQ, Life Events Questionnaire; MFQ-A, Mood and Feelings Questionnaire–Adult; MFQ-C, Mood and Feelings Questionnaire–Child; MFQ-P, Mood and Feelings Questionnaire–Parent on Child; PACE+, Patient-Centered Assessment and Counseling for Exercise Plus Nutrition; PBI, Parental Bonding Instrument; PHQ-A, Patient Health Questionnaire–adolescent version; RFQ-Y, Reflective Functioning Questionnaire for Youth; SCAS-C, Spence Children's Anxiety Scale; SCAS-P, Spence Children's Anxiety Scale–Parent; SHAPS, Snaith-Hamilton Pleasure Scale; WASI, Wechsler Abbreviated Scale of Intelligence; YSI-A, Youth Strengths Inventory–Adolescent; YSI-P, Youth Strengths Inventory–Parent*.

As shown in Table 3, there were no significant differences in the proportion of adolescents who self-identified as having white skin color across the three groups. School failure, drug use, and involvement in fights were less common in the LR group in comparison to both HR and MDD. Conversely, a history of running away from home was reported more frequently by those in the MDD group in comparison to both LR and HR. Adolescents in the LR group rated both their relationship with their father and between their parents more favorably than the adolescents in the HR and MDD groups. In terms of the relationship with mothers, there was a stepwise decrease from LR to HR to MDD—a similar pattern was observed for the proportion of adolescents who reported regularly meeting friends. Whereas all LR participants fell into the “no maltreatment” category, three quarters and almost all of those in the HR and MDD groups were classified, respectively, as having experienced “severe maltreatment.”

**Table 3:** IDEA-RS features in the IDEA-RiSCo sample.

	**Low risk (*n* = 50)**	**High risk (*n* = 50)**	**MDD (*n* = 50)**	**Group differences^b^**
	***n* (%)^a^**	***n* (%)^a^**	***n* (%)^a^**	
Sex, female	25 (50.00)	25 (50.00)	25 (50.00)	LR = HR = MDD
Skin color, non-white	22 (44.00)	26 (52.00)	26 (52.00)	LR = HR = MDD
Meets friends	49 (98.00)	40 (80.00)	30 (60.00)	LR > HR > MDD
School failure	0 (0.00)	29 (58.00)	25 (50.00)	LR < (HR = MDD)
Ran away	1 (2.00)	3 (6.00)	13 (26.00)	(LR = HR) < MDD
Any drug use	29 (58.00)	44 (88.00)	47 (94.00)	LR < (HR = MDD)
Fights	0 (0.00)	20 (40.00)	27 (54.00)	LR < (HR = MDD)
Relationship with father (mean, SD)	4.52 (0.79)	2.48 (1.22)	2.00 (1.18)	LR > (HR = MDD)
Relationship with mother (mean, SD)	4.78 (0.54)	3.92 (1.01)	3.14 (1.14)	LR > HR > MDD
Relationship between parents (mean, SD)	4.18 (1.08)	2.38 (1.23)	1.94 (1.04)	LR > (HR = MDD)
Childhood maltreatment				
None	50 (100.00)	1 (2.00)	0 (0.00)	LR > (HR = MDD)
Probable	0 (0.00)	12 (24.00)	4 (8.00)	LR < (HR > MDD)
Severe	0 (0.00)	37 (74.00)	46 (92.00)	LR < HR < MDD

a *Unless noted as mean (SD)*.

b *For a p < 0.05, comparisons between low risk (LR) vs. high risk (HR), LR vs. major depressive disorder (MDD), and HR vs. MDD, as indicated. “Relationship” variables were analyzed as continuous (mean, SD), with answers ranging = considered to range from 1 (bad) to 5 (great). IDEA-RiSCo, Identifying Depression Early in Adolescence Risk-Stratified Cohort*.

**Figure 8:**
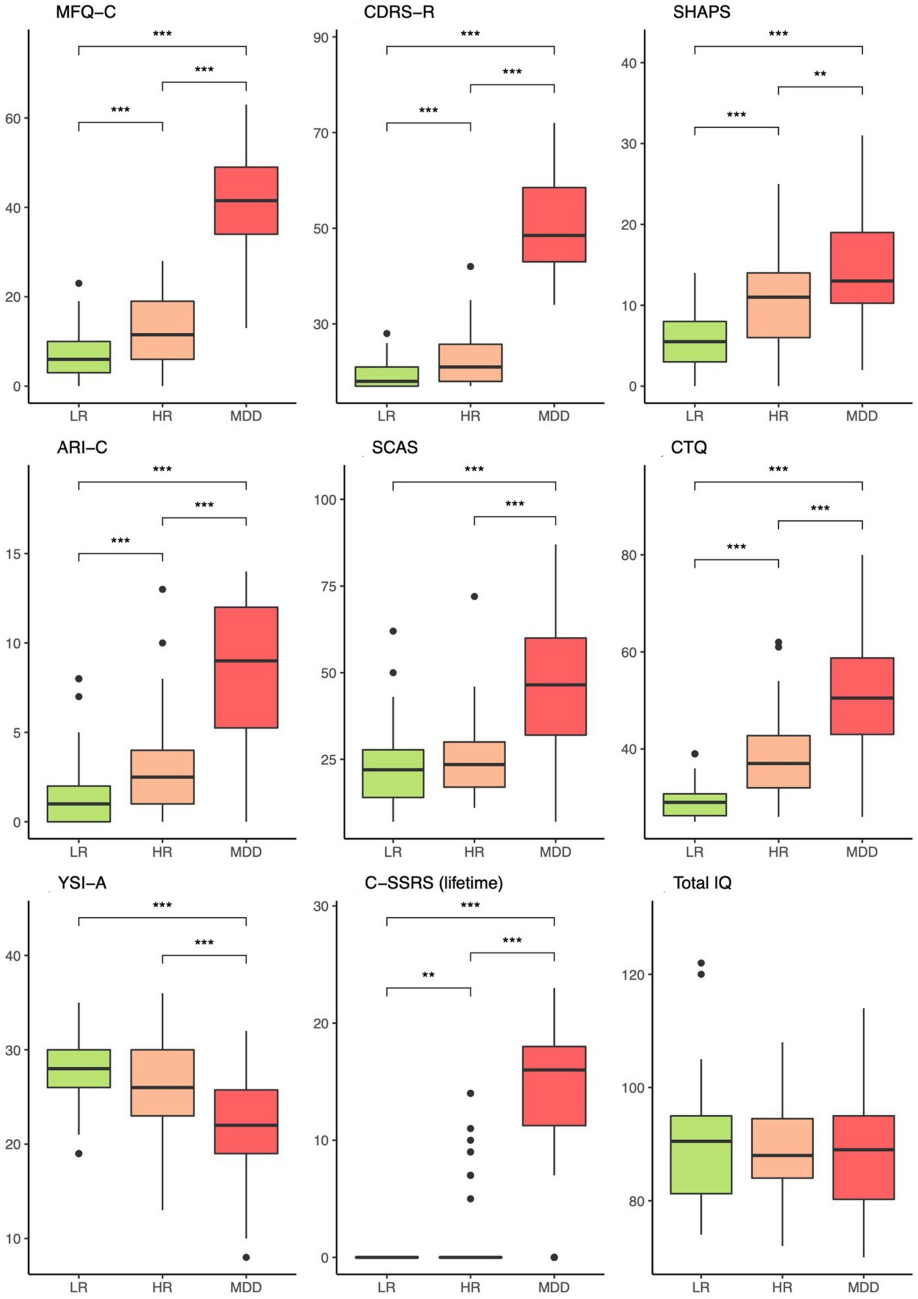
Phenotypic comparisons between groups based on reports by the adolescents. LR, low-risk; HR, high-risk; MDD, major depressive disorder; MFQ-C, Mood and Feelings Questionnaire—Child; CDRS-R, Children's Depression Rating Scale Revised; SHAPS, Snaith-Hamilton Pleasure Scale; ARI-C, Affective Reactivity Index—Child; SCAS-C, Spence Children's Anxiety Scale; CTQ, Child Trauma Questionnaire; YSI-A, Youth Strengths Inventory—Adolescent; C-SSRS, Columbia-Suicide Severity Rating Scale. CDRS-R ratings were based on information from both adolescent and caregiver, with priority to the former. The non-parametric Kruskal-Wallis test was applied to compare the means. **p < 0.01, ***p < 0.001.

**Figure 9:**
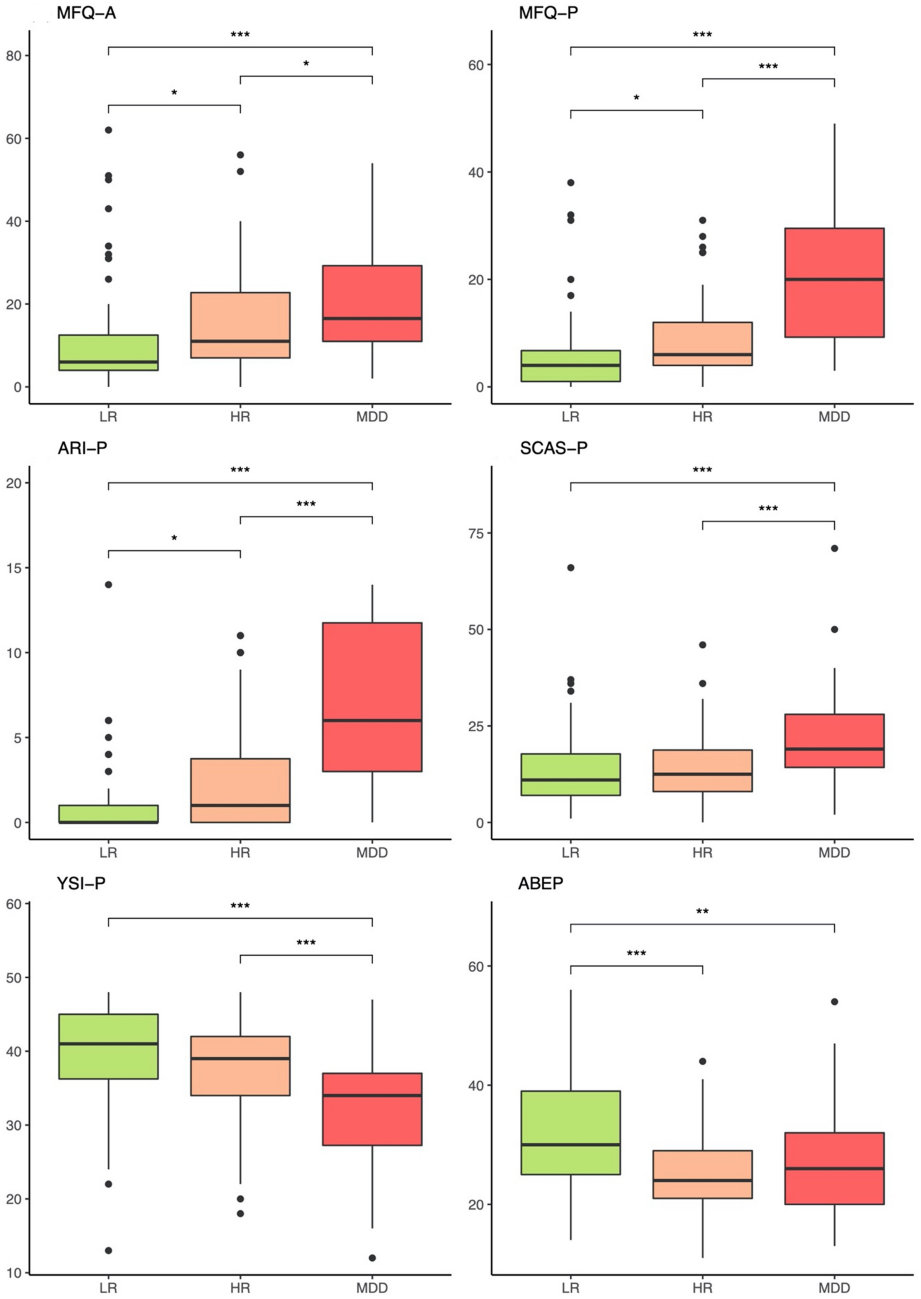
Phenotypic comparisons between groups based on reports by the caregivers. LR, low-risk; HR, high-risk; MDD, major depressive disorder; MFQ-A, Mood and Feelings Questionnaire—Adult (caregiver self-report); MFQ-P, Mood and Feelings Questionnaire—Parent (caregiver report on adolescent); ARI-P, Affective Reactivity Index—Parent; SCAS-P, Spence Children's Anxiety Scale—Parent; YSI-P, Youth Strengths Inventory—Parent; ABEP, Brazil socioeconomic classification index. The non-parametric Kruskal-Wallis test was applied to compare the means. *p < 0.05, **p < 0.01, ***p < 0.001.

Figures 8, 9 exhibit the results of phenotypic measures in the three groups based on reports by adolescents and primary caretakers, respectively. As shown in the figures, there was a stepwise increase from LR to HR to MDD across a variety of phenotypic measures: adolescent-reported (MFQ-C) and clinician-rated (CDRS-R) depressive symptomatology, clinical impression (CGI), and overall functioning (CGAS), as well as in specific measures of anhedonia (SHAPS) and irritability (ARI-C). A pattern in which the MDD group differed from both LR and HR groups emerged in relation to adolescent-rated suicidality (C-SSRS), anxiety (SCAS-C), insomnia (ISI), and positive attributes (YSI-A); as well as in caregiver-rated depression (MFQ-P), irritability (ARI-P), anxiety (SCAS-P), and positive attributes (YSI-P). This was also observed for the presence of any anxiety disorder (22, 26, and 56%) and any comorbid disorder (28, 36, and 62%) for the LR, HR, and MDD groups, respectively. Further details are provided in Table 2, Supplementary Material 9.

Participants in the MDD and HR groups had an elevated load of family history of depression in comparison to the LR group (Table 2). There was a stepwise decrease from MDD to HR to LR in terms of reporting childhood traumatic experiences (CTQ). Adolescents in the MDD group also reported more recent negative events (LEQ) in comparison to HR and LR; no differences in regard to neutral and positive events were observed. Both MDD and HR families exhibited lower socioeconomic scores (ABEP) in comparison to those in the LR group. The three groups did not significantly differ in terms of IQ scores and body mass index.

### Qualitative Interviews

Adolescents in the MDD group included in the qualitative analysis reported their perspectives on receiving a diagnosis of depression and participating in the IDEA-RiSCo study. The last participants included in the study (2 girls, 6 boys) were interviewed from October 2019 to December 2019. Extracts of their accounts can be found in Box 1. Another two girls were unable to attend the second interview and therefore were not included in the current analyses.

Box 1 **“Some questions we have to think a lot about”: the experience of participating in the IDEA-RiSCo study.**

Overall, adolescents had a limited understanding of the purposes of the research. None of them explicitly reported knowing why they underwent several steps of data collection, but rather explained the purpose of the research as being linked to the idea of finding out if they had “problems” or “something wrong with them”:

“*I think [data was collected] so it can be analyzed, to look for similarities with other people who have something similar to me.” (boy, age 15)*

About the initial screening phase in schools, most of the interviewed adolescents reported that they were even minded when they answered the screening questionnaire. Others, however, expressed concerns about answering the questions: they mentioned that they wondered whether they should answer truthfully. The idea of participating in the research as a way of being helped and having feelings and difficulties acknowledged was also often expressed by participants. Helping other adolescents who may be struggling with depression was also mentioned as a great motivator for participating in the research:

“*It was interesting to participate because I felt that it could help someone.” (boy, age 14)*

“*In the start, I thought it was something that wasn't going anywhere, but it was something that ended up helping me a lot.” (girl, age 16)*

When participating in the evaluation at the Clinical Research Center, all adolescents reported that the clinical interview was the most difficult part of the process. They expressed that it was emotional and hard to remember some past events and talk about their feelings, and answering the scales also demanded sustained attention.

“*Some questions were more emotional, about things that happened. One or two were harder, were about traumas […] Then it gets sad having to talk again about what happened.” (boy, age 16)*

However, they also added that the process was positive, even therapeutic in its own right:

“*I think it was good to at least be able to talk a little, identify with the questions and to know that I'm probably… Going through some of these problems.” (boy, age 15)*

About having their blood taken, several expressed that they were nervous about it. However, the presence of the research team and the support provided to the adolescent throughout the whole process was described as a way to face the anxiety related to the procedure:

“*I liked the researchers that were in the room with me, they started to talk to me, so I felt more comfortable” (boy, age 14)*

As the last part of the clinical protocol included an MRI scan, all adolescents reported that it was the most challenging part of the protocol in the sense of procedures before and during the scan and the completion of the tasks. They also mentioned discomfort with the necessity of being still for the whole assessment and that the total length of the procedure made them tired.

“*It was… Tiresome. I almost slept. It is weird. They put you inside this machine to see your brain… [I felt] anxious.” (boy, age 16)*

IDEA-RiSCo, Identifying Depression Early in Adolescence Risk-Stratified Cohort.

## Discussion

In this article, we described the rationale and methods for the IDEA-RiSCo study. Using a previously developed composite score (the IDEA-RS), we devised a new, risk-stratified cohort to study neurobiological correlates of risk and presence of depression among adolescents. Up to now, most studies with high-risk groups have focused on single risk factors to characterize groups. Relying on an empirically generated composite score comprising 11 sociodemographic variables allowed us to characterize groups using a definition anchored in the simultaneous occurrence of a range of risk factors and separate non-cases into those at high and low risk of future depression (rather than unhelpfully lumping them together).

Our risk score was developed using data from the Pelotas 1993 Birth Cohort study and exhibited a good discriminative capacity for the identification of adolescents at risk for depression (similar for instance to the Framingham Risk Score) (3). Although originally generated in a sample of Brazilian adolescents, the IDEA-RS has been demonstrated to predict (74) depression in other settings around the globe. Even without information on all the original 11 variables, the score was able to parse beyond chance high- and low-risk adolescents when externally assessed in samples from Nepal, New Zealand, Nigeria, and the United Kingdom (13–15). For the IDEA-RiSCo study, we collected information using the exact same questions from the Pelotas cohort, observing some differences in the prevalence of specific risk factors between the Pelotas and Porto Alegre samples, which could be at least in part understood as a consequence of differences in terms of the size of the cities (300,000 vs. 1,400,000 inhabitants), year (2008 vs. 2018–9), and setting (birth cohort vs. school-based sample) of data collection. Although the average IDEA-RS was higher in Porto Alegre in comparison to Pelotas, there was a remarkable resemblance in terms of how each factor was related to the others, as demonstrated by the similarity of the network structure in both samples.

The IDEA-RS uses sociodemographic information to stratify for the risk of developing depression. Differently from other approaches more aligned with the concept of indicated prevention (75), our score does not rely on using subthreshold symptomatology to predict a full-blown syndrome. Using subsyndromal psychopathology to identify at-risk mental states can require training and extensive assessments (76, 77), being less suitable in general population contexts (78). Our approach also differs from many high-risk studies as the IDEA-RS does not contain information on family history of depression. Although this has been one of the most replicated risk factors in the literature (79), our score was developed to be easily collectable directly from adolescents (who are frequently unlikely to know sufficient details about family psychiatric history), without needing to engage caregivers, which can be burdensome in terms of screening procedures. Moreover, we also acknowledge that the probability of someone reporting a positive family history can be largely influenced by the probability of family members having access to services and to diagnostic assessment, something that can be highly variable, especially in low- and middle-income settings. Furthermore, we assessed the incremental value of adding information on maternal depression to the IDEA-RS in the Pelotas dataset, and no meaningful classification improvement was observed (the opposite [adding the IDEA-RS to a stratification based on history of maternal symptoms of depression], however, enhanced risk estimation) (13).

Whether and to what extent the IDEA-RS captures the liability conferred by having a positive family history of depression remains to be understood. Future analyses comparing the IDEA-RS with information from polygenic risk scores (PRS) could be one strategy to further disentangle this issue. There is some suggestion that adding PRS to traditional risk scores can improve classification, although this has not always been the case (80). Importantly, families usually share not only genetic, but also environmental backgrounds, and some of the familial influences on depression risk could have been captured by the family-related items in the IDEA-RS (e.g., relationship with and between parents).

Considering the multifactorial etiology of depression, multiple pathways to the susceptibility for developing the disorder are likely (5). Individuals with a positive family history of depression have twice as much risk of developing the disorder (81). Also, a recent PRS for depression demonstrated a 2.5-fold increase in risk when comparing the highest and lowest risk deciles (82). In the IDEA-RiSCo sample, sociodemographic information was used to stratify individuals for risk of developing depression. Taking into account the evidence on social and environmental influences on immune/inflammatory factors and brain structure and function (83–85), focusing on adolescents at low and high extremes might enhance our ability to identify neurobiological correlates of depression risk. Indeed, the magnitude of risk associated with the IDEA-RS does not appear to be inferior to what has been observed using other traditional stratification strategies. Using similar cut-offs in the Pelotas 1993 Cohort, a 15-year-old girl classified as HR (≥90th percentile), in comparison to one classified as LR ( ≤ 20th percentile), exhibited an 8.67 (95% CI 3.56–21.08) times increased odds for having depression at age 18 years. Additionally, none of the boys in the LR group had depression at age 18. Still, although efficient in terms of parsing extremes, the specific cut-offs chosen for assigning individuals to LR and HR strata are arbitrary and should be further assessed for clinical relevance in subsequent studies.

In this report, we also presented the baseline clinical characteristics of the IDEA-RiSCo sample. After an extensive school-based screening process to identify individuals at low and high risk for developing depression in adolescence, we were able to form three groups consistently distinct in a wide range of phenotypic characteristics. Across a variety of measures of psychopathology and exposure to negative events, there was a clear pattern in which either the MDD group or both the HR and the MDD groups exhibited worse indicators in comparison to the LR group. Importantly, the differences seen between the LR and HR groups underscore the importance of not lumping them together as a homogeneous group of “non-cases.”

Regarding the adolescents' perspectives on participating in the IDEA-RiSCo study, they highlighted the importance of several aspects of conducting research with adolescents. First, eliciting trust from adolescents is a crucial aspect of the process. When answering questionnaires in the school setting, adolescents reported contemplating lying on their answers. Moreover, adolescents stressed the positive role of the research team in this process of trust and self-disclosure, as well as their overall comfort during specific steps of the process. Our data suggest that it is essential for adolescent participation to ensure that the research is conducted in an adolescent-friendly manner—especially by providing comfort and trust. Understanding how to better communicate with adolescents about research purposes and design plus consulting with them in designing research studies is likely to be crucial to ensure adolescent engagement.

Among the strengths of our study is the careful phenotypic characterization of the three groups with marked differences in terms of exposure to risk factors and manifestation of symptomatology. The comprehensive clinical assessment procedures, including the use of gold-standard instruments to collect information both from the adolescent and their primary caregiver and generate best estimate diagnoses is also an asset of the IDEA-RiSCo. Given the episodic nature of depression, it is extremely relevant to ensure that individuals with past depression, but who are not in an active episode, are not wrongly classified as “at risk,” as well as to require “cases” to be in a currently active depressive episode at the time of the assessment. Furthermore, we only included participants not using psychotropic medications, thereby making the sample more homogeneous. Due to possible temporal fluctuations in depressive symptomatology, performing clinical and neurobiological collections on the same day can also be seen as advantageous; unfortunately, due to logistical reasons we were not able to standardize the time of day for collection, but there were no differences in group proportions in terms of participants who were assessed in the morning or in the afternoon. The sample size can also be seen as a possible limitation of our study, which we believe can be counterbalanced by focusing on more homogeneous groups and employing comprehensive clinical assessment procedures, which is not always the case in large samples that frequently rely only on short, self-reported measures. Targeting extreme groups, although potentially advantageous for the identification of neurobiological correlates, has the intrinsic drawback of reducing the external validity of findings to individuals in the middle range. Furthermore, the requirement of a high IDEA-RS for the MDD group included in our design to allow for direct comparisons with the HR group, although focusing on adolescents with depression and high degree of vulnerability, inevitably makes the former less representative of the overall population of youths with depression. Lastly, we will be able to overcome the present cross-sectional constraint of the study with follow-up assessments that are currently underway—which will be essential, for instance, to confirm that HR adolescents are indeed at increased risk (as opposed to an alternative interpretation, according to which they could be more resilient to the emergence of depression despite high loading of risk factors).

The use of an empirically-based composite score to stratify risk for developing depression is a promising strategy to better understand the neurobiological mechanisms on the path to depression onset. The fact that nine out of ten children and adolescents in the globe live in low- and middle-income countries (LMICs) makes conducting this study in a middle-income country such as Brazil even more compelling (86, 87). Moreover, there is support for the approach adopted here among adolescent mental health experts in LMICs, including the focus on many of the IDEA-RS factors and the use of risk calculators (88). The underrepresentation of large proportions of the globe's population in the scientific literature is evident in the field of child and adolescent mental health (86, 87, 89). We hope that the IDEA-RiSCo study, by using state of the art methods to further understand the neurobiological underpinnings of risk and presence of depression among adolescents, will contribute to closing this gap.

## Data Availability Statement

The original contributions presented in the study are included in the article/Supplementary Material, further inquiries can be directed to the corresponding author/s.

## Ethics Statement

The studies involving human participants were reviewed and approved by Brazilian National Ethics in Research Commission (CAAE 50473015.9.0000.5327). Written informed consent to participate in this study was provided by the participants' legal guardian/next of kin.

## Author Contributions

CK, AC, HLF, RK, BAK, TR, LAR, JRS, and VM conceptualized the study and/or wrote the grant funding it. CK, AC, PM, AV, MA, LB, SB, HLF, BAK, RK, TM, SP, JP, TR, LS, BV, ZZ, VZ, JRS, and VM developed the study protocol. CK, CB, AC, PM, RP, AV, LB, SB, HLF, BAK, TM, SP, JP, TR, FR, LS, BV, AW, LY, ZZ, VZ, JRS, and VM contributed to data collection, analyses, and/or management. CK, CB, AC, PM, RP, AV, MA, LB, SB, HLF, RK, BAK, TM, SP, JP, TR, LAR, FR, LS, BV, AW, LY, ZZ, VZ, JRS, and VM wrote or revised sections of the manuscript. All authors approved the final version of the manuscript.

## Conflict of Interest

VM has received research funding from Johnson & Johnson, a pharmaceutical company interested in the development of anti-inflammatory strategies for depression, but the research described in this paper is unrelated to this funding. LAR has received grant or research support from, served as a consultant to, and served on the speakers' bureau of Bial, Medice, Novartis/Sandoz, Pfizer, and Shire/Takeda in the last 3 years. The ADHD and Juvenile Bipolar Disorder Outpatient Programs chaired by him have received unrestricted educational and research support from the following pharmaceutical companies: Novartis/Sandoz, and Shire/Takeda. He has received travel grants from Shire/Takeda to take part in the 2018 American Psychiatric Association congress. He also receives authorship royalties from Oxford Press and ArtMed. The remaining authors declare that the research was conducted in the absence of any commercial or financial relationships that could be construed as a potential conflict of interest.
