# Supplementary material for: The Identifying Depression Early in Adolescence Risk Stratified Cohort (IDEA-RiSCo): Rationale, Methods, and Baseline Characteristics
Source: Front Psychiatry. 2021 Jun 21;12:697144. doi: 10.3389/fpsyt.2021.697144 (PMC8255472; doi:10.3389/fpsyt.2021.697144)
Supplement: Supplementary file 1 [file Data_Sheet_1.docx]

Supplementary Material

**Supplementary Material 1.** The Identifying Depression Early in Adolescence Risk Score (IDEA-RS)

Administration of the IDEA-RS questionnaire in the schools was performed using a coded, unidentified form distributed to students after information on name, date of birth, self-reported sex, self-reported race/skin color, handedness, and parental contact information had been collected. Questions were selected to match *ipsis litteris* the original phrasing used in the Pelotas 1993 Birth Cohort study. On average, less than 15 minutes were required for administration of the IDEA-RS. Students were allowed to ask clarification questions, but researchers were not allowed to review the form to check for completion (forms were considered to be “complete” and therefore valid when only one answer was provided for each question and all questions were answered).

| Sex: | Male/Female |
| --- | --- |
| Your skin color or race is:* | White/Yellow/Indigenous/Brown/Black |
| Do you meet your friends often to talk, play or do anything else? | No/yes |
| Have you ever failed a school grade? | No/yes |
| Have you ever run away from home? | No/yes |
| Have you ever tried cigarettes?** | No/yes |
| Have you ever tried alcohol?** | No/yes |
| Have you ever tried sniffing glue?** | No/yes |
| Have you ever tried sniffing solvents or ethyl chloride (EC)?** | No/yes |
| Have you ever tried marijuana?** | No/yes |
| Have you ever tried cocaine or crack?** | No/yes |
| Have you ever tried LSD or acid?** | No/yes |
| Have you ever tried ecstasy or molly?** | No/yes |
| Have you ever used weight loss pills?** | No/yes |
| Have you ever used tranquilizers or sleeping pills?** | No/yes |
| Have you ever used any drug? ** | No/yes |
| In the last year, did you get into any fight in which somebody got hurt? | No/yes |
| Would you say your relationship with your father is: | Great/Very good/Good/Regular/Bad |
| Would you say your relationship with your mother is: | Great/Very good/Good/Regular/Bad |
| Would you say the relationship between your father and mother is: | Great/Very good/Good/Regular/Bad |
| Have you ever been separated from your parents so that you had to stay with someone else?*** | No/yes |
| At home, have you witnessed fights with physical aggression between adults, or has any adult assaulted a child or teenager?*** | No/yes |
| Have you experienced not having enough food at home, or have you had to wear dirty or torn clothes because you had no other?*** | No/yes |
| Have you ever thought or felt that your parents wished you were never born?*** | No/yes |
| Have you ever thought or felt that someone in your family hated you?*** | No/yes |
| Have you ever been beaten by an adult in your family or by someone who was taking care hard enough to leave marks or hurt you?*** | No/yes |
| Has anyone ever tried to touch you in a sexual way, or tried to make you touch them against your will, threatening you or hurting you?*** | No/yes |

* Self-reported skin color following Brazilian official census categories. For analyses, two categories (white vs. non-white) were formed.

** Questions about any lifetime use of alcohol, tobacco, cannabis, cocaine, and inhalants were combined into one variable using the OR rule, generating a binary variable for analyses.

*** Responses to seven dichotomous questions regarding lifetime psychological, physical, and sexual abuse and/or neglect were combined into three categories: zero positive answers=none, 1 positive=probable, 2 or more answers=severe.

**Supplementary Material 2.** Criteria for the IDEA Risk Stratified Cohort sample composition

| **Phase** | **Inclusion criteria** | **Exclusion criteria** |
| --- | --- | --- |
| *School screening* | Enrollment in 8th to 11th grades  Age 14 to 16 years  Right-handedness | Absent from school on the day of both assessments  Inability to complete the screening questionnaire |
| *Phone invitation* | Completed school questionnaire | Metallic accessories  Clinical conditions*  Use of psychotropic medication over last 30 days  Use of anti-inflammatory medication over last 14 days |
| *Clinical interview* | IQ > 70  Post-pubertal status | Current or lifetime:  Bipolar disorder  Schizophrenia or a primary psychotic disorder  Autism spectrum disorder  Substance use disorder  Eating disorder  Post-traumatic stress disorder |

* Excluded clinical conditions: known brain malformations, epilepsy, recent traumatic brain injury, diabetes, cystic fibrosis, HIV, asthma, rheumatologic conditions such as rheumatoid arthritis, systemic lupus erythematosus, psoriasis, purpura, oncologic conditions such as cancer, lymphoma, leukemia, and severe neurodevelopmental disorders, any recent/active infection or active inflammatory process.

**Supplementary Material 3.** Ascertainment

**School screening**

A total of 230 general public state schools (not vocational or special education schools) existed in the city of Porto Alegre in 2018, corresponding to 37,257 students enrolled in the 8th, 9th, 10th, and 11th grades according to official data (encompassing around three quarters of all enrollments for these grades). Schools were chosen by convenience (i.e., proximity to the HCPA Clinical Research Center, although some large schools were also recruited in more distant areas of the city [Figure S3.1]) and subsequently contacted by telephone or face-to-face visit for presentation of the project and team. This was followed by an official visit to enroll the school and secure an appropriate room for questionnaire administration. Each school was visited at least three times: 1) school enrollment visit; 2) presentation of the project to students in their classrooms, distribution of parent information forms (PIF), and ascertainment of participant eligibility; and 3) paper and pencil questionnaire administration.

**Figure S3.1.** Schools visited in the city of Porto Alegre

**
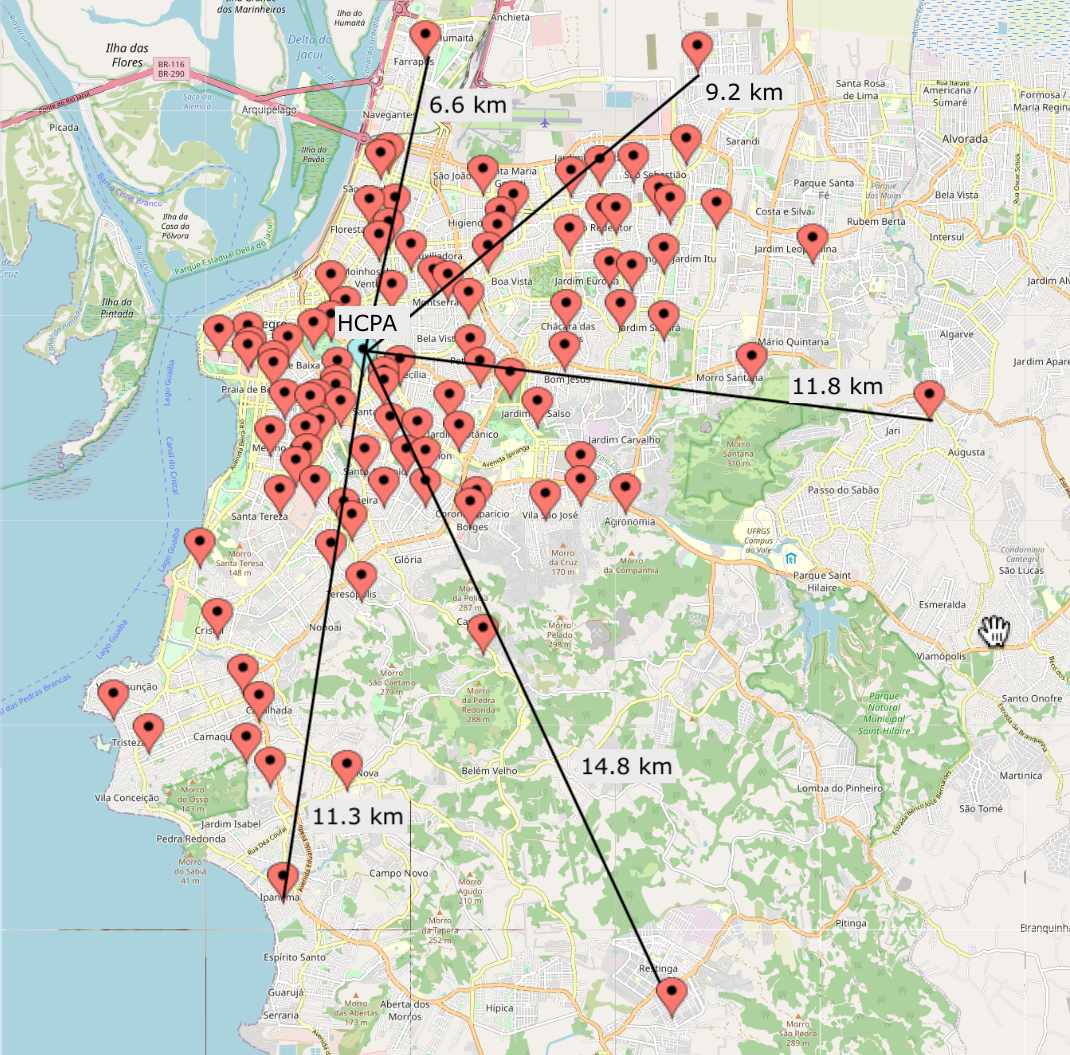
**

HCPA: Hospital de Clínicas de Porto Alegre.

School activities were performed in all cases by team researchers (mostly graduate or undergraduate students) trained for that purpose. On the day of PIF distribution, students were informed of the approximate date of data collection. The questionnaires were administered by two to four researchers (depending on the number of students) in a separate room designated by the school. Students who were unwilling or ineligible to participate remained in their usual classroom with the teacher. Students were only pulled out of the classroom if the teacher allowed them to go, and never during a test.

Of the 230 eligible schools, 126 were not contacted or visited because the required sample size had already been met (Figure S3.2); 104 agreed to participate, for a total of 24,559 students in grades 8 to 11. At each school, rolls for each class in the target grades were obtained from the Principal's office and examined to ascertain eligibility for each child. Thus, of the initial population of 24,559 students, 11,151 were excluded: 9,191 were out of age (<14 years or ≥17 years at distribution of the PIF); 1,439 were listed on the class roll at PIF distribution but had been transferred to another state school; 327 were on the class roll at PIF distribution but had cancelled enrollment and left the state school system; and 194 were excluded for cognitive impairment, language barriers, previous participation in the protocol at another school, or death. Therefore, 13,408 students were eligible for screening.

On the day of PIF distribution, 2,838 were absent and 41 refused the PIF; the PIF was thus distributed to 10,529 students who became eligible to complete the screening. Per protocol, administration of the questionnaire at the school took place at least 1 week after distribution of the PIF. At the first questionnaire round (Q1), 1,897 eligible students were absent, but were given the opportunity to complete the questionnaire at a later time (Q2). A further 1,769 students were excluded: 1,145 refused to participate; 408 were enrolled in three schools that engaged in a strike and were closed for questionnaires; and 216 students were not allowed participation by parents/guardians. Thus, 6,863 students completed Q1.

A minimum 7-day interval was again observed between Q1 and Q2. For logistic reasons, in 2019 only schools with at least 10 candidates for Q2 were revisited. That entailed exclusion of 364 students. At Q2, 514 students were absent, 154 refused to participate, and 8 did not have parental agreement. Thus, Q2 was administered to 857 students. Considering Q1 and Q2 administration, a total of 7,720 students completed screening questionnaires in 101 schools. To minimize typing errors, data were entered twice (Excel and REDCap) by researchers not involved in questionnaire administration, and reviewed by a separate team member for consistency and assessment of eligibility.

**Figure S3.2.** Flowchart of school and student inclusion


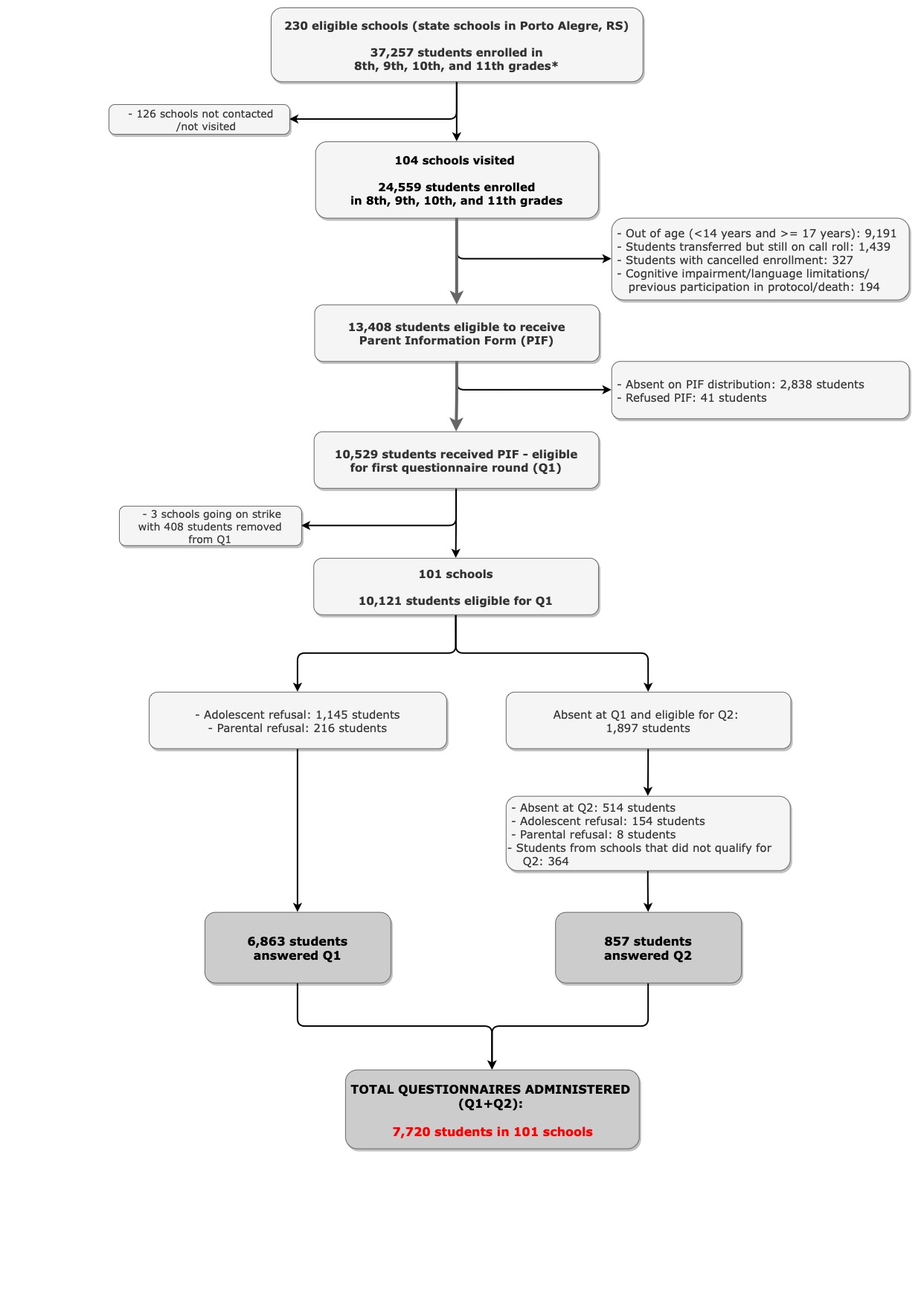


*Instituto Nacional de Estudos e Pesquisas Educacionais Anísio Teixeira. Sinopse Estatística da Edução Básica 2018. Brasília: Inep, 2019. Available at https://cidades.ibge.gov.br/brasil/rs/porto-alegre/pesquisa/13/78117. Accessed 28 January 2020.

**Ascertainment of eligibility and inclusion**

Of the overall group of 7,720 adolescents, 5,954 did not meet the inclusion criteria of right-handedness, no missing answers in the PHQ-A and IDEA-RS questionnaires, or classification into the LR, HR, or MDD groups. Thus, 1,766 were eligible for further assessment: 369 classified as LR, 389 as HR, and 1,008 as MDD.

Parents or guardians were contacted over the phone and invited to accompany the adolescent to the Hospital de Clínicas de Porto Alegre (HCPA). The number of exclusions at each step appears in Figure S4.3. Contact with 21, 81, and 506 participants in the LR, HR, and MDD groups respectively was not attempted because the target sample was met before they were called.

Additional exclusion criteria at this stage, ascertained over the phone, were as follows: wearing a metal device (e.g., braces); medical comorbidity preventing participation (asthma, brain malformation, cancer, lymphoma, leukemia; cystic fibrosis, diabetes, epilepsy, HIV, past concussion, cranioencephalic trauma, systemic lupus erythematosus, psoriasis, severe neurodevelopmental impairment, hypertension, active inflammatory process or other medical contraindications to take part in the proposed research procedures); and current use of psychotropic medications or other non-psychiatric drugs (anti-inflammatory drugs, inhaled corticosteroids, antibiotics) that could interfere with the results.

Among 348 LR participants with attempted contact, 72 were unreachable, 100 refused to participate, and 98 were excluded based on the telephone interview with parents/guardians: 84 wore a metal device (unable to undergo MRI), 5 had a medical comorbidity preventing participation, 3 were taking psychotropic medication, and 6 were taking other types of medication that could interfere with the results. Therefore, in the LR group, 78 adolescents were scheduled for clinical evaluation. Of these, 14 did not show up, and 64 LR adolescents underwent clinical evaluation.

In the HR group, contact was attempted with 308 families: 72 were unreachable, 71 refused to participate, 60 adolescents were excluded for wearing a metal device, 3 had a medical comorbidity preventing inclusion, 3 were taking psychotropic medication, and 2 were taking other medications that could interfere with the results. Therefore, in the HR group, 97 adolescents were scheduled for clinical evaluation – 34 missed the appointment, and 63 underwent clinical evaluation.

In the MDD group, contact was attempted with 502 families, of which 132 were unreachable and 82 refused participation. Additional exclusions were as follows: 88 adolescents wearing a metal device, 8 with medical comorbidities, 18 taking psychotropic medication, 5 taking other medication that could interfere with the results, and 3 excluded for other reasons. Therefore, in the MDD group, 166 adolescents were scheduled for clinical assessment – 33 missed the appointment, and 133 underwent clinical evaluation.

**Figure S3.3.** Flowchart of clinical, laboratory, and imaging exclusion/inclusion steps


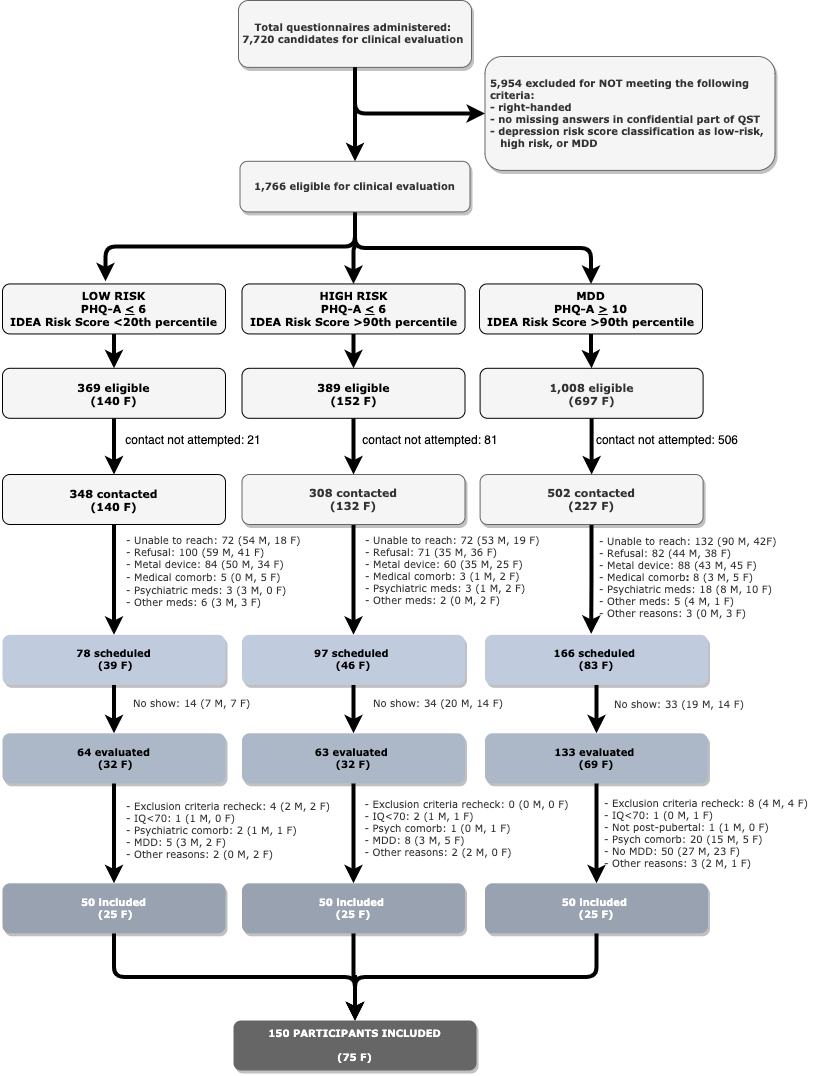


**Group eligibility assignment**

Further exclusions at the moment of clinical evaluation were as follows: in the LR group, 4 were excluded by criteria recheck, 1 had IQ<70, 2 had exclusionary psychiatric comorbidities, 5 were diagnosed with MDD, and 2 were excluded for other reasons (1 informed to be wearing a metal device before the MRI scan and 1 refused blood collection). In the HR group, 2 were excluded for IQ<70, 1 had a psychiatric comorbidity, 8 had MDD, and 2 were excluded for other reasons (unable to complete the MRI scan). In the MDD group, 8 were excluded following criteria recheck, 1 had IQ<70, 1 did not have post pubertal status, 20 had psychiatric comorbidities, 50 did not have their MDD diagnosis confirmed, and 3 were excluded for other reasons (1 informed to be wearing a metal device before the MRI scan, 2 did not complete MRI). In the absence of lifetime history of a depressive episode for the LR and HR groups and presence of a current depressive episode diagnosis for the MDD group, adolescents were invited to provide biological samples.

**Supplementary Material 4.** Translation and adaptation of instruments

Translation of the Adapted Resilience Scale (ARS) (1, 2), the Patient-Centered Assessment and Counseling for Exercise Plus Nutrition (PACE+) (3) and Life Events Questionnaire (LEQ) (4) was performed by the research team following the steps proposed by Harkness (5) for questionnaire translation and assessment, i.e., Translation, Review, Adjudication and Documentation (TRAPD). The TRAPD system uses a structured translation approach that does not involve back-translation. In TRAPD, translation is a team effort that relies on translators, reviewers and adjudicators. The following steps are involved: 1) initial parallel translation by two translators independently of each other; 2) review by a third team member who is knowledgeable about the aims of project and skilled in questionnaire development; a review of the technical aspects of the translation to ensure that coding structures are correct should also be performed; and 3) adjudication of translation adequacy by another team member on the basis of an analysis of the translations, comments made by the reviewer and their own professional expertise. Each step is documented in detail to provide an audit trail describing the translation process for analysis where there are conceptual nuances on particular questions/answers. The same procedure was used to modify the currently available version of the Patient Health Questionnaire (PHQ-9) in Brazilian Portuguese (6) to include the adaptations proposed for the adolescents population (PHQ-A) (7).

**Supplementary Material 5.** MRI data acquisition parameters

High-resolution structural images were collected with a T1 weighted MPRAGE volumetric acquisition in the sagittal plane with TR=8.7ms, TE=4ms, TI=900ms, flip angle=8^o^, field of view=256 x 242mm, 200 slices with 0.94mm thickness, acquisition matrix 272 x 255, resulting in a 0.94 x 0.94 x 0.94mm^3^ voxel resolution. Blood oxygenation level dependent signal (BOLD) images were collected with the following acquisition parameters: TR=2000ms, TE=30ms, flip angle=90^o^, in transaxial plane aligned to the anterior and posterior commissure line, field of view=240 x 240mm, 36 slices with 3.5mm thickness, slice gap 0.35mm and acquisition matrix 80 x 80. An Esys fMRI by Invivo with a 32” screen and console system was used to present stimuli for tasks using E-prime Runtime version 3.0.

**Supplementary Material 6.** Task-fMRI preprocessing

First, BOLD images were realigned to the first volume to correct for head motion. Next, the mean BOLD image was co-registered to the high-resolution anatomical image. The anatomical image was segmented and spatially normalized into standard stereotactic space (Montreal Neurological Institute [MNI] template) using a 12‐parameter affine model (final resolution of functional images = 2 mm isotropic voxels). The warps were applied to the functional images to normalize these into MNI space. BOLD images were smoothed with a Gaussian kernel, set at 6‐mm full‐width at half‐maximum.

**Supplementary Material 7.** Three-year depression risk in Porto Alegre and Pelotas according to sex and IDEA-RS density

**
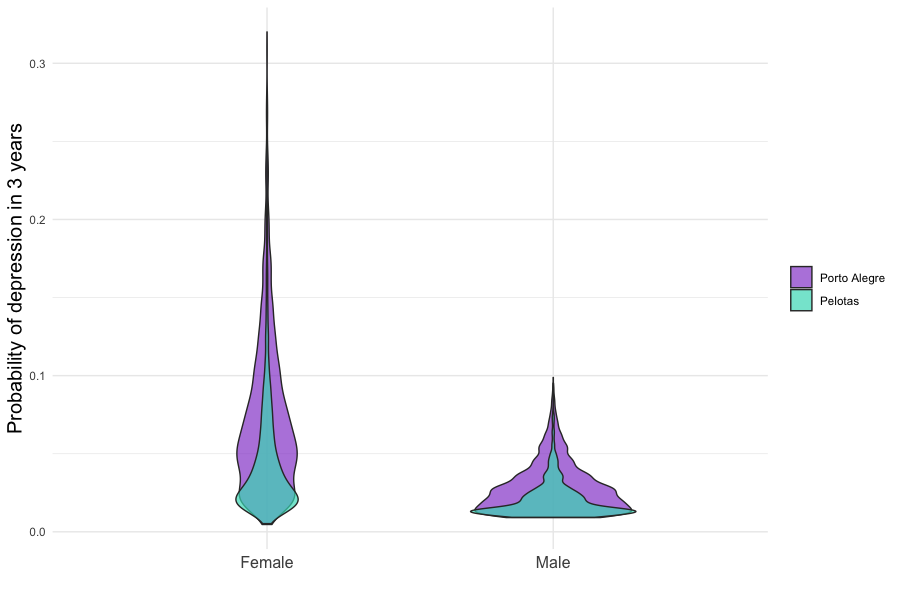
**
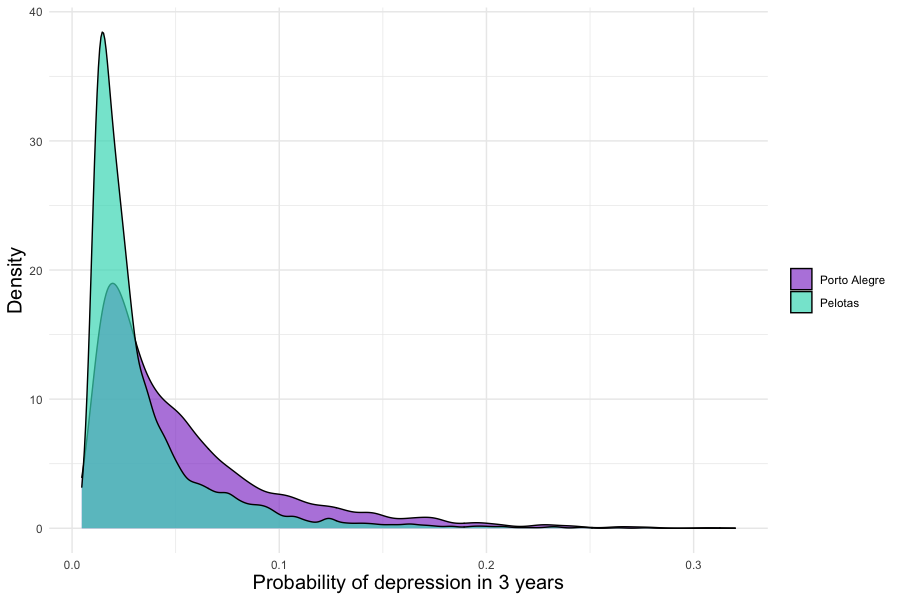


IDEA-RS: IDEA Risk Score. Distribution of the IDEA-RS by sex and sample. In figure A, the pattern of distribution is similar in both samples when divided by sex. There are more girls with higher IDEA-RS, making the plot longer, whereas there are more boys with lower IDEA-RS, making the bottom of the plot wider. Figure B shows a higher proportion (in %) of adolescents with lower IDEA-RS in Pelotas compared to Porto Alegre.

**Supplementary Material 8.** Network analyses

Our aim was to assess the networks of variables included in the IDEA-RS in Pelotas and Porto Alegre in order to compare their structure in both samples (8). The networks were conducted on R Studio 3.6.1 (R Foundation for Statistical Computing, Vienna, Austria), using the “bootnet” package (8) and “mgm” method for estimating networks based on Mixed Graphical Model. This model aims to look at each interaction between variables as unique and not explained away by other (mediating) variable (9). Also, different types of variables have been able to be analyzed together, such as categorical, dichotomized, continuous and discrete variables.

We performed undirected networks, which analyze the mutual relationship between variables. Each node represents a variable; variables are connected by undirected and weighted edges. The edges represent the association between variables; the width of edges is proportional to the absolute value of the underlying parameter. Positive associations are represented by green edges, whereas negative associations are red. The nodes are positioned using the Fruchterman-Reingold algorithm (“spring” layout) which organizes the nodes with the greatest number of connections in the center of the graph.

We calculated four centrality measures (10-13) to assess the connectedness between variables:

- **Node strength** reflects the involvement of a node within the network and is calculated as the sum of the number and strength of all connections of one node to all other nodes.
- **Closeness** gives an indication of how easy it is to reach all other nodes from the node of interest. It is inversely related to the sum of all shortest path lengths from one node to all other nodes and it.
- **Betweenness** indicates how well one node connects other nodes. It is related to how often a node is in the shortest paths between other nodes.
- **Expected influence** can be used to identify highly influential nodes in the network. It computes node strength without taking the absolute value of edge-weights.

**Figure S8.1.** Centrality measures for the IDEA-RS items in Pelotas and Porto Alegre


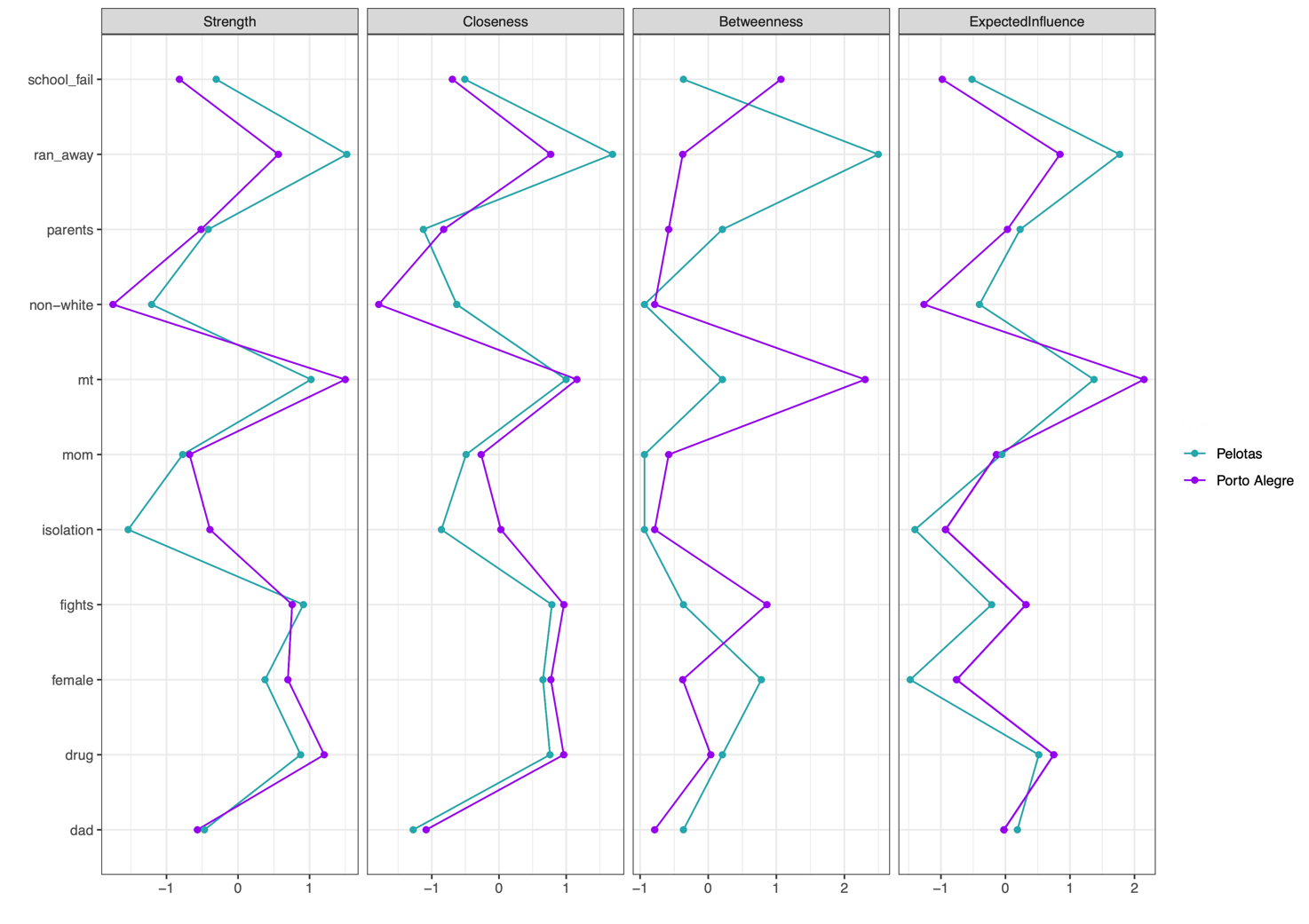
 Considering standardized z-score values, the variable of maltreatment (mt) had high indexes in the two samples, but was higher in Porto Alegre. Also, in Porto Alegre, the variable of history of involvement in fight had high indexes. In Pelotas, the variable of history of running away (ran_away) from home had the highest indexes. This means that these variables are more relevant in the model. Non-white: non-white skin color; drug: any drug use; school_fail: history of school failure; isolation: social isolation; fights: fight involvement; mom: relationship with mother; dad: relationship with father; parents: relationship between parents; mt: childhood maltreatment; ran_away: ran away from home.

In order to compare networks of IDEA-RS variables in both Pelotas and Porto Alegre samples, we used the Network Comparison Test (NCT) (14). NCT makes it possible to compare two (independent) networks based on overall global strength and network structure. The results indicate that the type of association (positive or negative) is similar in the two networks for most of the variables, differing only in their strength. The difference in overall global strength (connectivity) between the networks was S = 0.18. This indicates that although Pelotas has a higher global strength (S = 6.57) compared to Porto Alegre (S = 6.39), this difference is not significant (p = 0.9). Also, the value of the maximum difference in edge weights (structure) of the observed networks was M = 0.23 (p = 0.28). This reveals that the relation between variables in both networks seems to be similar, indicating that the samples are comparable.

**Supplementary Material 9.** Comorbidities

Psychiatric diagnoses were assessed using the Schedule for Affective Disorders and Schizophrenia for School-Age Children-Present and Lifetime Version (K-SADS-PL) (15). There were no differences between the groups except for generalized anxiety disorder and separation anxiety disorder, as well as for any anxiety disorder, and any comorbid disorder; in all these cases, a higher prevalence was observed in MDD, while LR and HR were similar.

**Table S9.1** Lifetime comorbid diagnoses for the IDEA-RiSCo sample

|  | Low risk  (n=50) | High risk (n=50) | MDD  (n=50) | Group differences^a^ |
| --- | --- | --- | --- | --- |
|  | n (%) | n (%) | n (%) |  |
| Any comorbid disorder | 14 (28.00) | 18 (36.00) | 31 (62.00) | (LR = HR) < MDD |
| Any anxiety disorder | 11 (22.00) | 13 (26.00) | 28 (56.00) | (LR = HR) < MDD |
| Generalized anxiety disorder | 1 (2.00) | 3 (6.00) | 15 (30.00) | (LR = HR) < MDD |
| Separation anxiety disorder | 1 (2.00) | 3 (6.00) | 14 (28.00) | (LR = HR) < MDD |
| Specific phobias | 5 (10.00) | 7 (14.00) | 10 (20.00) |  |
| Social anxiety disorder | 4 (8.00) | 2 (4.00) | 9 (18.00) |  |
| Panic disorder | 1 (2.00) | 0 (0.00) | 1 (2.00) |  |
| Attention-deficit/hyperactivity disorder | 2 (4.00) | 5 (10.00) | 3 (6.00) |  |
| Obsessive-compulsive disorder | 0 (0.00) | 0 (0.00) | 3 (6.00) |  |
| Enuresis | 0 (0.00) | 3 (6.00) | 1 (2.00) |  |
| Oppositional defiant disorder | 0 (0.00) | 2 (4.00) | 1 (2.00) |  |
| Agoraphobia | 0 (0.00) | 0 (0.00) | 0 (0.00) |  |
| Disruptive mood dysregulation disorder | 0 (0.00) | 0 (0.00) | 0 (0.00) |  |
| Conduct disorder | 0 (0.00) | 0 (0.00) | 0 (0.00) |  |
| Encopresis | 0 (0.00) | 0 (0.00) | 0 (0.00) |  |

^a^For a p < 0.05, comparisons between low risk (LR) vs. high risk (HR), LR vs. major depressive disorder (MDD), and HR vs. MDD, as indicated. As bipolar, schizophrenia/psychotic, autism spectrum, substance use disorder, eating and post-traumatic stress disorders were exclusion criteria for all groups, no included individual met criteria for these diagnoses.

**References**

1. Wagnild GM, Young HM. Development and psychometric evaluation of the Resilience Scale. J Nurs Meas. 1993;1(2):165-78.

2. Kohrt BA, Worthman CM, Adhikari RP, Luitel NP, Arevalo JMG, Ma J, et al. Psychological resilience and the gene regulatory impact of posttraumatic stress in Nepali child soldiers. Proc Natl Acad Sci U S A. 2016;113(29):8156-61.

3. Patrick K, Sallis JF, Prochaska JJ, Lydston DD, Calfas KJ, Zabinski MF, et al. A multicomponent program for nutrition and physical activity change in primary care: PACE+ for adolescents. Arch Pediatr Adolesc Med. 2001;155(8):940-6.

4. Kiddle B, Inkster B, Prabhu G, Moutoussis M, Whitaker KJ, Bullmore ET, et al. Cohort Profile: The NSPN 2400 Cohort: a developmental sample supporting the Wellcome Trust NeuroScience in Psychiatry Network. Int J Epidemiol. 2018;47(1):18-9g.

5. Harkness JA. Questionnaire translation. Cross-cultural survey methods. 2003;1:35-56.

6. Fraguas R, Jr., Henriques SG, Jr., De Lucia MS, Iosifescu DV, Schwartz FH, Menezes PR, et al. The detection of depression in medical setting: a study with PRIME-MD. J Affect Disord. 2006;91(1):11-7.

7. Johnson JG, Harris ES, Spitzer RL, Williams JBW. The patient health questionnaire for adolescents: validation of an instrument for the assessment of mental disorders among adolescent primary care patients. J Adolesc Health. 2002;30(3):196-204.

8. Epskamp S, Borsboom D, Fried EI. Estimating psychological networks and their accuracy: A tutorial paper. Behav Res Methods. 2018;50(1):195-212.

9. Haslbeck JMB, Waldorp LJ. Estimating Time-Varying Mixed Graphical Models in High-Dimensional Data. J Stat Software. 2020;93(8):1-46.

10. Barrat A, Barthélemy M, Pastor-Satorras R, Vespignani A. The architecture of complex weighted networks. Proc Natl Acad Sci U S A. 2004;101(11):3747-52.

11. Boccaletti S, Latora V, Moreno Y, Chavez M, Hwang DU. Complex networks: Structure and dynamics. Physics Reports. 2006;424(4):175-308.

12. Opsahl T, Agneessens F, Skvoretz J. Node centrality in weighted networks: Generalizing degree and shortest paths. Soc Networks. 2010;32(3):245-51.

13. Robinaugh DJ, Millner AJ, McNally RJ. Identifying highly influential nodes in the complicated grief network. J Abnorm Psychol. 2016;125(6):747-57.

14. van Borkulo CD. Symptom network models in depression research: From methodological exploration to clinical application [Ph.D. Thesis]: University of Groningen; 2018.

15. Caye A, Kieling RR, Rocha TB, Graeff-Martins AS, Geyer C, Krieger F, et al. Schedule for Affective Disorders and Schizophrenia for School-Age Children - Present and Lifetime Version (K-SADS-PL), DSM-5 update: translation into Brazilian Portuguese. Braz J Psychiatry. 2017;39(4):384-6.
